# Exploring midwifery role and scope in acute early pregnancy care: a survey of midwives and midwifery students in Australia

**DOI:** 10.1186/s12884-025-07567-3

**Published:** 2025-04-16

**Authors:** Nicole Freeman, Tracey Moroney, Jane Warland, Kate Cheney, Michelle Hobday, Zoe Bradfield

**Affiliations:** 1 School of Nursing, Faculty of Health Sciences, Curtin University, Perth, WA Australia; 2 Women’s and Newborn’s Health Service, King Edward Memorial Hospital, Perth, WA Australia; 3 Faculty of Health and Medical Sciences, The University of Adelaide, Adelaide, South Australia Australia; 4 Department of Nursing and Midwifery Education and Research, Women’s and Newborn’s Health Service, King Edward Memorial Hospital, Perth, WA Australia; 5 Faculty of Medicine and Health, The University of Sydney, Sydney, NSW Australia; 6 PO Box 245, Wembley, WA 6913 Australia

**Keywords:** Early pregnancy, Maternity care, Midwifery, Pregnancy complications, Professional role, Scope of practice

## Abstract

**Background:**

The scope of practice of the contemporary midwife encompasses a range of sexual and reproductive healthcare, including care throughout pregnancy. Midwives are experts in pregnancy care, but many do not provide care for women with unexpected (acute) complications in early pregnancy (< 20 weeks) in Australia. Women experiencing acute pregnancy complications < 20 weeks usually attend an emergency department rather than a maternity unit. These settings do not typically employ midwives and may not meet women’s need for timely and informed physical care and psychosocial support. A gap in evidence exists in relation to the role and scope of practice of the midwife in acute early pregnancy care provision in Australia.

**Methods:**

Midwives and midwifery students in Australia were purposively sampled and invited to complete an online cross-sectional survey exploring midwifery practice in acute early pregnancy care. Data were collected from July 1st to September 30th, 2024. Quantitative data were analysed using descriptive and inferential statistics. Free-text responses were analysed using inductive content analysis.

**Results:**

Responses from 294 midwives and 46 midwifery students (*n* = 340) were analysed. Participants recognised that midwives should provide acute care in early pregnancy, and many had knowledge, confidence or experience in this area. The most reported setting for acute early pregnancy care provision was the general emergency department; early pregnancy assessment service models were also common. Some settings prioritised the employment of registered nurses over midwives in acute early pregnancy services. Challenges to midwives providing acute early pregnancy care included inadequate clinical exposure as qualified midwives, and women being placed in non-maternity settings.

**Conclusion:**

Participants supported midwives providing acute care in early pregnancy, confirming that midwives’ professional scope should not be impacted by pregnancy gestation or outcome. However, current midwifery education programs may not be adequately preparing midwives to provide comprehensive care for women with acute early pregnancy complications. Those midwives who are providing care may not be fulfilling professional scope. Findings have utility in supporting policy, education and service review, and highlight further gaps in evidence for future research.

**Supplementary Information:**

The online version contains supplementary material available at 10.1186/s12884-025-07567-3.

## Background

Midwives are primary clinicians in maternity care settings, with a professional image aligned with the provision of care across the childbearing continuum [1]. In reality, a midwife’s role and scope of practice, both in Australia and internationally, is multi-faceted and encompasses a wide spectrum of reproductive and sexual health care including the care of women before, during and after pregnancy [2, 3]. The term midwife has protected title status in Australia and other countries [4, 5], and midwives must practice in accordance with national codes, guidelines, and standards unique to the midwifery profession [3, 4, 6, 7, 8]. The philosophical focus of midwifery acknowledges both the profound experience and significance of pregnancy and childbearing for women and families, and the need for midwives to partner with and support women throughout this time [9].

Despite professional recognition that midwives are experts in the provision of care throughout pregnancy [2, 3], many midwives do not provide care for women in *early pregnancy*, typically defined in Australian clinical practice and published evidence as under 20 weeks gestation [10, 11]. Women who experience *acute* complications requiring unscheduled care in early pregnancy (e.g., bleeding, pain, severe vomiting) may attend a non-maternity setting such as a gynaecology service or an emergency department (ED) that primarily employs registered nurses (RNs) [12–15]. Although midwives in some Australian and international settings are caring for women with acute early pregnancy complications [16–18], it remains an area of pregnancy care often provided by RNs [13, 19, 20]. Early Pregnancy Assessment Service (EPAS) models that provide specialist assessment, support and follow-up for clinically stable women with early pregnancy complications may employ midwives but are also commonly staffed by RNs [21, 22]. The potential for midwives to provide comprehensive physical and emotional care to women with a range of acute early pregnancy complications [23] is likely unrealised.

Women have reported a variety of challenges when accessing acute care in early pregnancy [24, 25]. Complications, including threatened or actual early pregnancy loss, can be physically and psychologically distressing for women and partners [24–27]. Interactions with care providers can have a significant impact on the care experience, and influence women’s ongoing psychological health [25, 28]. Settings like an ED can be chaotic and lack privacy, with long waiting times that may exacerbate women’s feelings of anxiety or distress [14, 26, 29]. These services may not employ clinicians who have specialist pregnancy knowledge and insight regarding the information and support that women require [27]. Women and partners have clearly reported that, in addition to sensitive and compassionate physical care, they need their concerns and emotional needs acknowledged by attending staff [15, 26, 27]. In a busy ED with competing clinical interests, the provision of timely, informative, and emotionally comprehensive care for women in early pregnancy can be challenging [16]. Ongoing follow up and information after an initial acute presentation may be poorly co-ordinated, focusing on physical aspects of care and lacking continuity and psychosocial support, particularly in the context of bereavement or loss [27].

Midwife standards for practice in Australia [6] include supporting women’s access to maternity care and promoting the potential for midwives to improve health outcomes for women. Whilst there is important acute early pregnancy research from other health professionals both nationally and internationally [24, 25, 26], there are gaps in evidence regarding midwives’ role and scope in acute early pregnancy care provision in Australia [16]. Examples from Australian research with midwifery authorship describe acute early pregnancy care from the largely unchallenged perspective of it being embedded in nursing practice [10, 14, 20, 30]. Internationally, there has been some midwifery-led research, including in countries where midwives do provide acute care in early pregnancy [23, 31, 32]. However, as is the case in Australia, much of the international research in this area is led by other health professionals including nurses, doctors and psychologists [15, 33, 34].

The aim of this study was to explore midwives and midwifery students’ perspectives regarding the role and scope of practice of midwives in acute early pregnancy care provision in Australia. The study forms part of a larger research project and is some of the first midwife-led Australian research undertaken in this area of practice. Study findings will provide novel benchmarking data regarding the role and scope of practice of midwives in the acute early pregnancy care period, informing further exploration of this area of midwifery practice and pregnancy care in Australia.

## Methods

An exploratory cross-sectional survey design, which involves gathering information at a single point in time from a group of participants [35], was chosen for this study. Cross-sectional surveys provide a useful ‘snapshot’ [36] of the research topic and have utility to gather important and benchmarking evidence from busy clinicians [37].

Midwifery practice in Australia is regulated at a national level and midwives must be registered to practice and use the title midwife [5, 38]. Although most midwives are employed in hospital settings, they also practice in community settings and private continuity models, as well as in non-clinical roles including research and management [39]. A small percentage of midwives in some hospital settings also work in continuity models [39]. Whilst midwifery was traditionally studied after gaining a nursing qualification in Australia, undergraduate midwifery education was introduced in 2002 [40]. Consequently, an increasing number of midwives registering in Australia each year are not RNs [41].

*Endorsed midwives*, subject to the requirements of local, state or territory legislation, are authorised to prescribe a range of scheduled medicines and order diagnostic testing for women in Australia [42]. The upward trend in suitably experienced midwives undertaking additional education and training to gain regulatory endorsement further extends the scope capacity of the midwife as an autonomous practitioner in a range of settings [43].

### Data collection and analysis

A total of 340 midwives and midwifery students recruited from around Australia participated in this online survey exploring midwifery role and scope of practice in the provision of acute care in early pregnancy (< 20 weeks gestation).

### Survey development

The role and scope of practice of midwives in this area of pregnancy care has not previously been explored. Survey items were therefore developed using existing evidence as well as author expertise in midwifery, survey development, acute early pregnancy care, and scope of practice. As there is little existing evidence on midwifery role and scope in acute early pregnancy care in Australia, data from earlier research [16, 44] provided valuable insight to support survey tool development. Statistician input in survey planning and development strengthened this process.

The survey consisted of demographic questions and multiple choice response items designed to measure participants’ self-reported knowledge, confidence, and perspectives regarding midwifery practice in acute early pregnancy care [35, 45] (Additional file 1). There was one concluding open-ended question for participants to share additional thoughts. The survey was intentionally designed with both discrete choice and free-text responses to reduce survey fatigue amongst busy clinicians and facilitate complete responses. To address face and content validity [46] survey content was reviewed by acute early pregnancy clinicians, midwives without early pregnancy expertise, and a consumer representative with lived experience of acute early pregnancy complications. Online survey functionality and usability was piloted with selected midwives and academics using mobile and desktop devices, with final minor adjustments made prior to the survey launch [47]. Pilot responses were not included for analysis.

### Sampling and recruitment

The target sample population was midwives registered with the Nursing and Midwifery Board of Australia (NMBA), and midwifery students enrolled in a pre-registration program in Australia. Knowledge or experience in acute early pregnancy care was not required. Participants were recruited via social media, email, displayed flyers, and in-person contact, from July 1st to September 30th, 2024. The Qualtrics (USA, 2024) platform was used to host the survey, accessed via a secure, confidential, anonymous link. The IP address of the participant was recorded. A participant information form was hosted on the survey splash page and consent was provided through a hurdle question to confirm eligibility. Demographic data was collected, but no identifying information was requested.

An exploratory cross-sectional survey sample is not intended to represent all potential participants within a population [48]. However the aim during recruitment for this study was to achieve a varied sample that reflected the *characteristics* of the midwifery profession in terms of demographics, experience, and practice settings [49]. This would enable some inferences to be drawn from the findings that may be relevant or applicable to other midwifery contexts in Australia and internationally [50]. In keeping with this type of exploratory study design, calculation of a sample size was not required. A recent study of midwives in Australia, hosted in Qualtrics and recruiting for a similar timeframe, reported over 330 valid responses [37]. It was anticipated a similar number would be recruited for this study.

### Data analysis

Survey data were imported from Qualtrics into the statistical software program SPSS (IBM, v29, 2023). Records (*n* = 30) were excluded from analysis if participants had not progressed beyond demographic questions. Incomplete surveys (*n* = 38) accounted for most of the missing data in included records; valid percentages were reported for those variables with missing data.

Quantitative data were analysed using descriptive and inferential statistics. Descriptive statistics were undertaken for each survey item. Some response items were condensed from four-point scales into binary categories to facilitate analysis. The two-tailed Chi-Square Test (χ^2^) of Independence was used to test for differences between proportions in sample sub-groups; a p-value of = < 0.05 was considered statistically significant (α = 0.05) [51]. When the assumptions for a Chi-Square Test were violated (e.g., cell count < 5) a Fishers Exact Test was used instead.

Qualitative data from open-ended text responses were imported into NVivo (v14, 2023) software and analysed using *inductive content analysis* [52–54]. Content analysis enables the researcher to organise and make sense of narrative data through a systematic approach to reducing and grouping data into meaningful categories [52, 53]. When a research topic is relatively unexplored or knowledge is fragmented [52] as is the case in this study, an *inductive* approach to analysis enables concepts and categories to be developed based on the study data, rather than on pre-existing theory or knowledge [52, 53]. Applying a well-respected analytical process to qualitative data in this study enabled the reporting of diverse and individual participant perspectives not necessarily captured in the closed-response survey questions [54].

### Findings

There were 340 survey responses included in the final analysis. Midwives (*n* = 294, 86%) and midwifery students (46, 14%) participated in the survey (Table 1). Sample characteristics aligned with Australian demographic midwifery data for 2023/24 [43], except for midwife age, with higher percentages of midwives in the 35–54 year age groups in this study compared with national data. Midwives and midwifery students from all states and territories of Australia, working in metropolitan and rural settings, participated with the largest number from Western Australia (127, 38%), the home state of the primary study authors.

**Table 1:** Participant characteristics

Characteristic		Midwives *n* (%^+^)	Students *n* (%^+^)	Total *n* (%^+^)
Professional status		294 (86)	46 (14)	340 (100)
Other professional qualifications^^ Endorsed Midwife Registered Nurse IBCLC Lactation Consultant Maternal Child Health/ Child Health Other		70 (24) 167 (49) 30 (9) 26 (8) 19 (6)	N/A 10 (3) Nil Nil 19 (6)	70 (24) 177 (52) 30 (9) 26 (8) 38 (12)
Age (years) 18–24 25–34 35–44 45–54 55–64 65+		5 (2) 56 (16) 78 (23) 77 (23) 69 (20) 9 (3)	21 (6) 12 (4) 11 (3) 1 (< 1) 1 (< 1) Nil	26 (8) 68 (20) 89 (26) 78 (23) 70 (20) 9 (3)
Gender Woman or female Man or male Non-binary *or* another term *or* prefer not to answer		287 (84) 3 (1) 4 (1)	46 (14) Nil Nil	333 (98) 3 (1) 4 (1)
Aboriginal or Torres Strait Islander No Aboriginal Torres Strait Islander		289 (85) 4 (1) 1 (< 1)	43 (13) 3 (1) Nil	332 (98) 7 (2) 1 (< 1)
Language other than English at home No Yes		280 (83) 14 (4)	38 (11) 8 (2)	318 (94) 22 (6)
State or Territory of usual practice New South Wales Victoria Queensland South Australia Western Australia Tasmania Northern Territory Australian Capital Territory		45 (13) 49 (14) 31 (9) 39 (11) 114 (34) 3 (1) 6 (2) 7 (2)	3 (1) 12 (4) 11 (3) 2 (1) 13 (4) 1 (< 1) 1 (< 1) 3 (1)	48 (14) 61 (18) 42 (12) 41 (12) 127 (38) 4 (1) 7 (2) 10 (3)
Location of Practice Metropolitan Larger Regional Smaller Rural Remote Community		201 (59) 50 (15) 37 (11) 6 (2)	38 (11) 4 (1) 4 (1) Nil	239 (70) 54 (16) 41 (12) 6 (2)
Qualification completed/being completed for registration Hospital-based certificate Undergraduate degree Dual degree Postgraduate Diploma Postgraduate Masters		52 (15) 87 (26) 20 (6) 116 (34) 19 (5)	Nil 23 (7) 13 (4) 4 (1) 6 (2)	52 (15) 110 (33) 33 (10) 118 (35) 25 (7)
Highest post-secondary education Diploma or certificate Postgraduate Diploma Bachelor’s Degree Masters PhD None		20 (6) 72 (21) 97 (29) 88 (26) 15 (4) N/A	14 (4) 3 (1) 15 (4) 2 (1%) Nil 12 (4%)	34 (10) 75 (22) 112 (33) 90 (27) 15 (4) 12 (4)
Learnt or learning about AEPC as a midwifery student Yes No Unsure		175 (52) 75 (22) 44 (13)	26 (8%) 15 (4%) 5 (1%)	201 (60) 90 (26) 49 (14)
Clinical placements in AEPC as a midwifery student^ Yes No Unsure		78 (23) 210 (62) 6 (2)	11 (3%) 25 (7%) 8 (2%)	89 (26) 235 (69) 14 (4)
Additional midwife sample (*n* = 294) characteristics (n; %^++^)				
Employment sector Public Private Private Practice Higher education Other	221 (75) 22 (8) 31 (11) 13 (4) 7 (2)	**Current model of care** Continuity Core/non-continuity Rotational hospital Education, Research, Academia Management or Executive Other		77 (26) 109 (37) 37 (12) 34 (12) 23 (8) 14 (5)
Years registered in Australia 5 years or less 6–10 years 11–15 years 16–20 years 21 + years	51 (17) 66 (23) 54 (18) 44 (15) 79 (27)	**Experience in acute early pregnancy** Never provided Previously provided Currently providing		91 (31) 96 (33) 107 (36)

^+^Percentage of whole sample; ^++^Percentage of midwife sample; **^**Percentages add up to > 100% as participants could select more than one response; **^^**Percentages add up to < 100% due to missing data; AEPC: Acute early pregnancy care

Participants held qualifications in midwifery and other disciplines including public health, education, and management. Just over half (177, 52%) were also RNs. For midwives, the most common qualification completed for initial registration was a Postgraduate Diploma (116, 35%). In contrast, only 4 midwifery students (9%) were undertaking this qualification. One third of all participants had completed or were undertaking undergraduate studies in midwifery (110, 33%); most of these were midwives registered < 20 years, or midwifery students.

Midwife participants were employed in public sector (221, 75%), private sector (22, 8%), or private practice (31, 11%) services, working in continuity (77, 26%) and non-continuity (146, 49%) models as well as in education, research and management (57, 20%). Others worked in aeromedical retrieval, outreach programs, and shared care models. Endorsed midwives accounted for 24% of midwife participants (*n* = 70), representing 5% of all midwives with endorsement registered in Australia in 2023/24 [43].

### Exposure to acute early pregnancy care education and practice

A large proportion of midwife participants received acute early pregnancy care education as midwifery students (175, 60%), but significantly less had clinical placements as students in early pregnancy settings (78, 27%; χ^2^ p = < 0.001). Similar trends were reported for midwifery students (26, 57% v 11, 24% respectively; *p* =.054). Despite lower levels of clinical exposure as students, many midwives (203, 69%) reported having experience in acute early pregnancy care once qualified. Midwives who had a clinical placement as midwifery students more frequently reported subsequent experience as qualified midwives (65, 83% v 13, 17%; χ^2^ p = < 0.001), as did midwives registered for more than ten years (141, 69% v 62, 31%; χ^2^ p = < 0.001), and those working in non-metropolitan locations (76, 82% v 127, 63%; χ^2^*p* =.001). A higher percentage of endorsed midwives reported they had experience in acute early pregnancy care (56, 80% v 147, 66%; χ^2^*p* =.023), and worked in a non-metropolitan setting (32, 46% v 61, 27%; χ^2^*p* =.004) than non-endorsed colleagues.

**Fig. 1:**
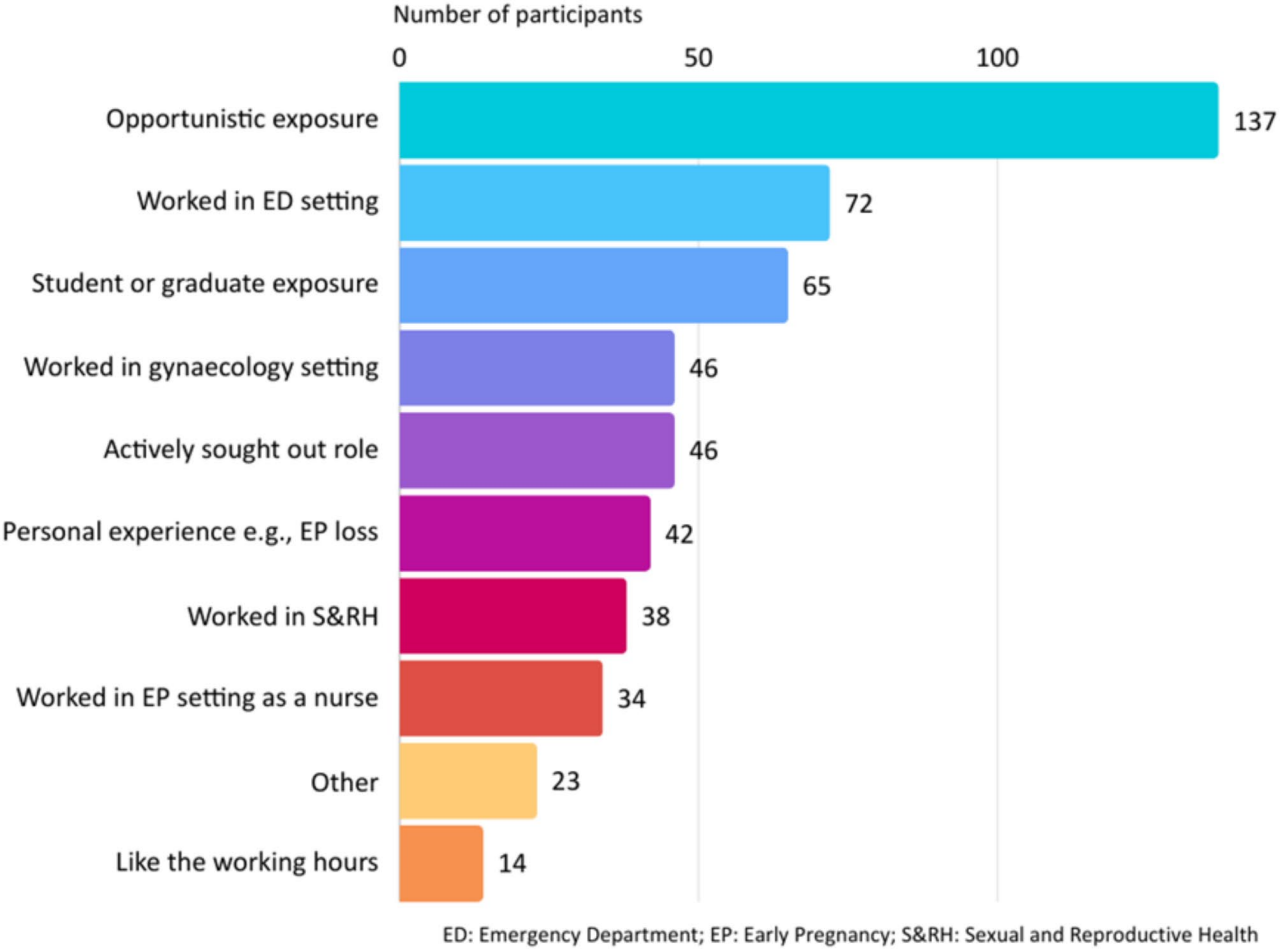
Midwives motivations and enablers to work in acute early pregnancy care

Most midwives and midwifery students not currently providing acute early pregnancy care reported they would be interested in working in this area of practice (201, 87%). For those midwives with experience, the chance to work in this area of practice was most commonly via opportunistic or chance exposure (137, 67%). Just over one third (72, 35%) had worked in an ED that provided care for women in early pregnancy, with two-thirds (48, 67%) of these midwives also RNs. Other considerations included working in a rural setting or in a continuity model of care (Fig. 1). A higher proportion of midwives who were RNs reported acute early pregnancy experience from working in gynaecology (30, 65% v 16, 35%) and sexual and reproductive health (22, 60% v 16; 42%) settings compared to non-RN peers.

### Knowledge and confidence regarding acute early pregnancy complications and care

Participants were asked to rate their *knowledge* of miscarriage, ectopic pregnancy and hyperemesis gravidarum (HG), and the care and management of these conditions (Additional file 2). Participants reported strong (fair or strong) levels of knowledge for miscarriage and HG more frequently compared to ectopic pregnancy. A higher proportion of participants who were RNs reported strong knowledge of miscarriage care (129, 75% v 96, 62%, χ^2^*p* =.017), ectopic pregnancy (130, 75% v 92, 60%, χ^2^*p* =.003), ectopic pregnancy care (114, 66% v 75, 49%, χ^2^*p* =.002), and HG care (139, 80% v 105, 68%, χ^2^*p* =.012) than non-RN participants. The number of participants reporting stronger knowledge of clinical care provision and management of miscarriage, ectopic pregnancy and HG was consistently lower than reported theoretical knowledge of the conditions (Table 2).

**Table 2:** Participants’ theoretical and clinical care knowledge of selected acute early pregnancy complications

Early pregnancy complication	Fair or strong knowledge *n* (%^#^)	Limited or no knowledge *n* (%^#^)	*p*-value
Miscarriage *n* = 327^ Theoretical knowledge of condition Knowledge of clinical care provision	272 (83.2) 225 (68.8)	55 (16.8) 102 (31.2)	< 0.001*
Ectopic pregnancy *n* = 327^ Theoretical knowledge of condition Knowledge of clinical care provision	222 (67.9) 189 (57.8)	105 (32.1) 138 (42.2)	< 0.001*
Hyperemesis Gravidarum *n* = 327^ Theoretical knowledge of condition Knowledge of clinical care provision	277 (84.7) 244 (74.6)	50 (15.3) 83 (25.4)	< 0.001*

^#^Valid percentages; **^**Participant numbers vary due to missing data

Participants were asked to rate their *level of confidence* to provide aspects of acute early pregnancy care (Table 3). Most participants (248, 76%) reported confidence (i.e., strong or fair confidence) to identify when women with acute early pregnancy complications required referral to psychological or social care services. Participants frequently reported confidence to assess and treat pain (213, 65%) and recognise/respond to clinical deterioration (210, 64%). While 64% (209) of participants reported confidence regarding support and education for early pregnancy loss (e.g., miscarriage), only a third (107, 33%) were confident to provide this support for women with ectopic pregnancy. Fewer participants reported confidence to administer the folic acid antagonist methotrexate [55] for medical management of ectopic pregnancy (81, 25%) and assess beta human chorionic gonadotrophin (b-hCG) levels (154, 47%).

**Table 3:** Participants reported confidence to provide acute care < 20 weeks gestation

Clinical example	Strongly or fairly confident^ *n* (%^#^)	Limited or no confidence^ *n* (%^#^)
Physical care provision		
Assess and organise analgesia for a woman experiencing acute pain	213 (65.1)	114 (34.9)
Recognise and respond to clinical deterioration in a woman with heavy vaginal bleeding	210 (64.2)	117 (35.8)
Manage the third stage of labour for a woman who has miscarried	166 (50.9)	160 (49.1)
Perform a speculum examination on a woman with vaginal bleeding	160 (48.9)	167 (51.1)
Identify an acceptable upward trend in beta-hCG^+^ levels	154 (47.1)	173 (52.9)
Administer methotrexate for ectopic pregnancy	81 (24.8)	245 (75.2)
Psychosocial care provision		
Identify when a woman with an acute early pregnancy complication should be offered referral to psychosocial services	248 (75.8)	79 (24.2)
Provide support and education for a woman with early pregnancy loss	209 (63.9)	118 (36.1)
Provide information regarding community resources available for support and information following early pregnancy loss	185 (56.6)	142 (43.4)
Explain to a junior doctor the treatment options for a missed miscarriage	155 (47.4)	172 (52.6)
Explain to a woman the treatment options and post operative support for a tubal ectopic pregnancy	107 (32.7)	220 (67.3)

**^**Condensed response options; ^#^Valid percentages; ^+^Human chorionic gonadotrophin

A higher proportion of qualified midwives, and those registered as a midwife > 10 years, reported confidence across all items. Midwives working in rural settings reported confidence to manage the third stage of labour following miscarriage (60, 66% v 103, 53%; χ^2^*p* =.037) and recognise when women should be offered referral for psychosocial care (80, 88% v 53, 78%; χ^2^*p* =.047) more frequently than metropolitan peers. Endorsed midwives more frequently reported confidence across several items (Table 4).

**Table 4:** Reported confidence levels of endorsed and non-endorsed midwives to provide acute care < 20 weeks

Clinical example	Strongly or fairly confident^ *n* (%^#^)	Limited or not confident^ *n* (%^#^)	*p*-value
Physical care provision			
Assess and organise analgesia for a woman experiencing acute pain: Endorsed Midwife *n* = 70 Midwife but not endorsed *n* = 217	48 (68.6) 156 (71.9)	22 (31.4) 61 (28.1)	*p* =.594
Perform a speculum examination on a woman with vaginal bleeding: Endorsed Midwife *n* = 70 Midwife but not endorsed *n* = 217	42 (60.0) 112 (51.6)	28 (40.0) 105 (48.4)	*p* =.221
Recognise and respond to clinical deterioration in a woman with heavy vaginal bleeding: Endorsed Midwife *n* = 70 Midwife but not endorsed *n* = 217	50 (71.4) 154 (71.0)	20 (28.6) 63 (29.0)	*p* =.941
Administer methotrexate for ectopic pregnancy: Endorsed Midwife *n* = 70 Midwife but not endorsed *n* = 217	19 (27.1) 58 (26.9)	51 (72.9) 158 (73.1)	*p* =.962
Identify an acceptable upward trend in beta-hCG^+^ levels: Endorsed Midwife *n* = 70 Midwife but not endorsed *n* = 217	44 (62.9) 99 (45.6)	26 (37.1) 118 (54.4)	*p* =.012*
Manage the third stage of labour for a woman who has miscarried: Endorsed Midwife *n* = 70 Midwife but not endorsed *n* = 217	42 (60.0) 121 (56.0)	28 (40.0) 95 (44.0)	*p* =.559
Psychosocial care provision			
Provide support and education for a woman with early pregnancy loss: Endorsed Midwife *n* = 70 Midwife but not endorsed *n* = 217	52 (74.3) 148 (68.2)	18 (25.7) 69 (31.8)	*p* =.336
Explain to a woman the treatment options and post-operative support for a tubal ectopic pregnancy: Endorsed Midwife *n* = 70 Midwife but not endorsed *n* = 217	33 (47.1) 72 (33.2)	37 (52.9) 145 (66.8)	*p* =.035*
Provide information regarding community resources that offer support and information following early pregnancy loss: Endorsed Midwife *n* = 70 Midwife but not endorsed *n* = 217	50 (71.4) 125 (57.6)	20 (28.6) 92 (42.4)	*p* =.039*
Identify when a woman should be offered referral to psychological or social care services: Endorsed Midwife *n* = 70 Midwife but not endorsed *n* = 217	58 (82.9) 175 (80.6)	12 (17.1) 42 (19.4)	*p* =.681
Explain to a junior doctor the treatment options for first trimester missed miscarriage: Endorsed Midwife *n* = 70 Midwife but not endorsed *n* = 217	45 (64.3) 106 (48.8)	25 (35.7) 111 (51.2)	*p* =.024*

^Condensed response categories; ^#^Percentage of endorsed or non-endorsed midwives; ^+^Human chorionic gonadotrophin

### Scope of practice and setting of care in early pregnancy

Participants reported their level of agreement with a series of statements relating to midwives’ scope of practice in acute early pregnancy care, and the setting in which care is provided. Two additional statements relating to workplace scope of practice guidance were completed by midwife participants (Table 5).

**Table 5:** Level of agreement regarding scope of practice and setting of care

Scope of practice statement	Agree or strongly agree^ *n* (%^#^)	Disagree or strongly disagree^ *n* (%^#^)
Midwives’ scope of practice in Australia includes the care of women with acute complications < 20 weeks	263 (84.0)	50 (16.0)
Midwives’ scope of practice in Australia includes the care of women experiencing pregnancy loss < 20 weeks	267 (85.3)	46 (14.7)
Midwives practising in Australia should provide care to non-pregnant women with acute reproductive health concerns	209 (66.8)	104 (33.2)
Midwives are the best qualified professionals to provide acute care for pregnant women < 20 weeks	270 (86.3)	43 (13.7)
Registered nurses who are NOT midwives have the professional and educational preparation to provide acute early pregnancy care	71 (22.7)	241 (77.3)
A woman experiencing a threatened miscarriage < 20 weeks should be cared for in a maternity not emergency or gynaecological setting	256 (82.6)	56 (17.4)
Women requiring treatment for HG^+^<20 weeks should be cared for in a maternity rather than an emergency or gynaecological setting	277 (88.5)	36 (11.5)
Women experiencing early pregnancy loss *should be* cared for separate from other pregnant and postnatal women	272 (86.9)	41 (13.1)
Women experiencing early pregnancy loss *want to be* cared for separate from other pregnant and postnatal women	276 (88.2)	37 (11.8)
Women recovering from ectopic surgery *should be* cared for in a gynaecology or surgical setting rather than a maternity setting	206 (65.8)	107 (34.2)
**Additional statements for midwives only**		
Where I currently work, there are clinical guidelines or policies that clearly outline what *midwives* can and cannot do as part of their role	166 (60.4)	109 (39.6)
Where I currently work, there are clinical guidelines or policies that clearly outline what *colleagues* can and cannot do as part of their role	142 (51.8)	135 (48.2)

# Percentage of all participants; ^Condensed response categories; ^+^Hyperemesis Gravidarum

Most participants agreed that midwives are the best qualified professionals to provide acute care in early pregnancy (270, 86%), and that midwifery scope in Australia includes the care of women with early pregnancy complications (263, 86%). This was a significant finding with midwife participants registered for more than 10 years (147, 90% v 87, 78%; χ^2^*p* =.007), or with experience in acute early pregnancy care (169, 89% v 65, 77%; χ^2^*p* =.010), when compared with midwives registered less than ten years, or with no acute early pregnancy experience respectively. A lower proportion of endorsed midwives agreed that clear workplace guidance existed regarding the role and scope of midwives (33, 49% v 133, 64%; χ^2^*p* =.021), and of other colleagues (28, 41% v 114, 55%; χ^2^*p* =.043), compared with non-endorsed peers. Midwives with experience in early pregnancy care provision more frequently agreed that midwives scope includes the provision of care to women with acute reproductive concerns who are *not* pregnant (140, 73% v 50, 59%; χ^2^*p* =.017) than those without this experience. Less than a quarter of participants (71, 23%) many of whom were dual registered themselves, agreed that RNs who are *not* midwives have the professional preparation to provide acute early pregnancy care. However, more participants who were RNs agreed that nurses had the necessary preparation for this area of pregnancy care (44, 28% v 19, 17%; χ^2^*p* =.033) compared to non-RN participants.

The majority of participants agreed that women requiring treatment for HG (277, 89%), or threatened miscarriage (256, 82%), should be cared for in a maternity setting, although 87% (272) also agreed women experiencing events such as early pregnancy loss might *should* and would *want to be* cared for separately from other pregnant and postnatal women. In contrast, two thirds of participants (206, 66%) agreed women recovering from operative treatment of ectopic pregnancy should be cared for in a non-maternity (e.g., surgical) setting.

### Acute early pregnancy settings in Australia

**Fig. 2:**
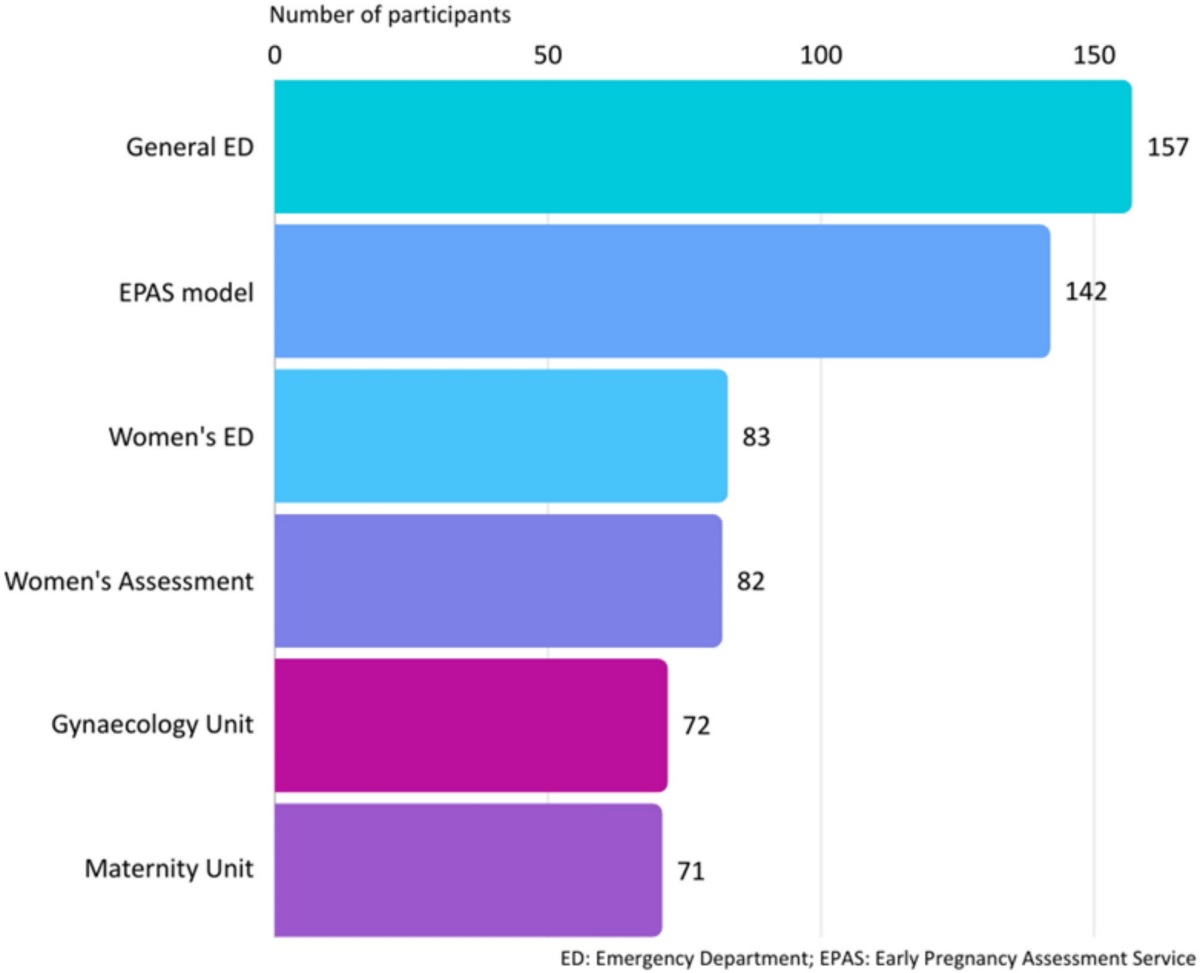
Participant workplace acute care service options for women < 20weeks

Participants were asked *where care was provided* in their workplace for pregnant women with acute complications < 20 weeks (Fig. 2). Nearly half of participants worked in a health service where women received care in a general ED (157, 46%), with this more commonly reported by rural participants (69, 68% v 88, 37%). Many participants (142, 42%), particularly those working in metropolitan settings (126, 53%) reported an EPAS-type service was available where they worked. Nearly one-quarter of participants (83, 24%) worked in a setting that provided care in a women’s ED; nearly all of these (80, 96%) were in metropolitan locations.

**Fig. 3:**
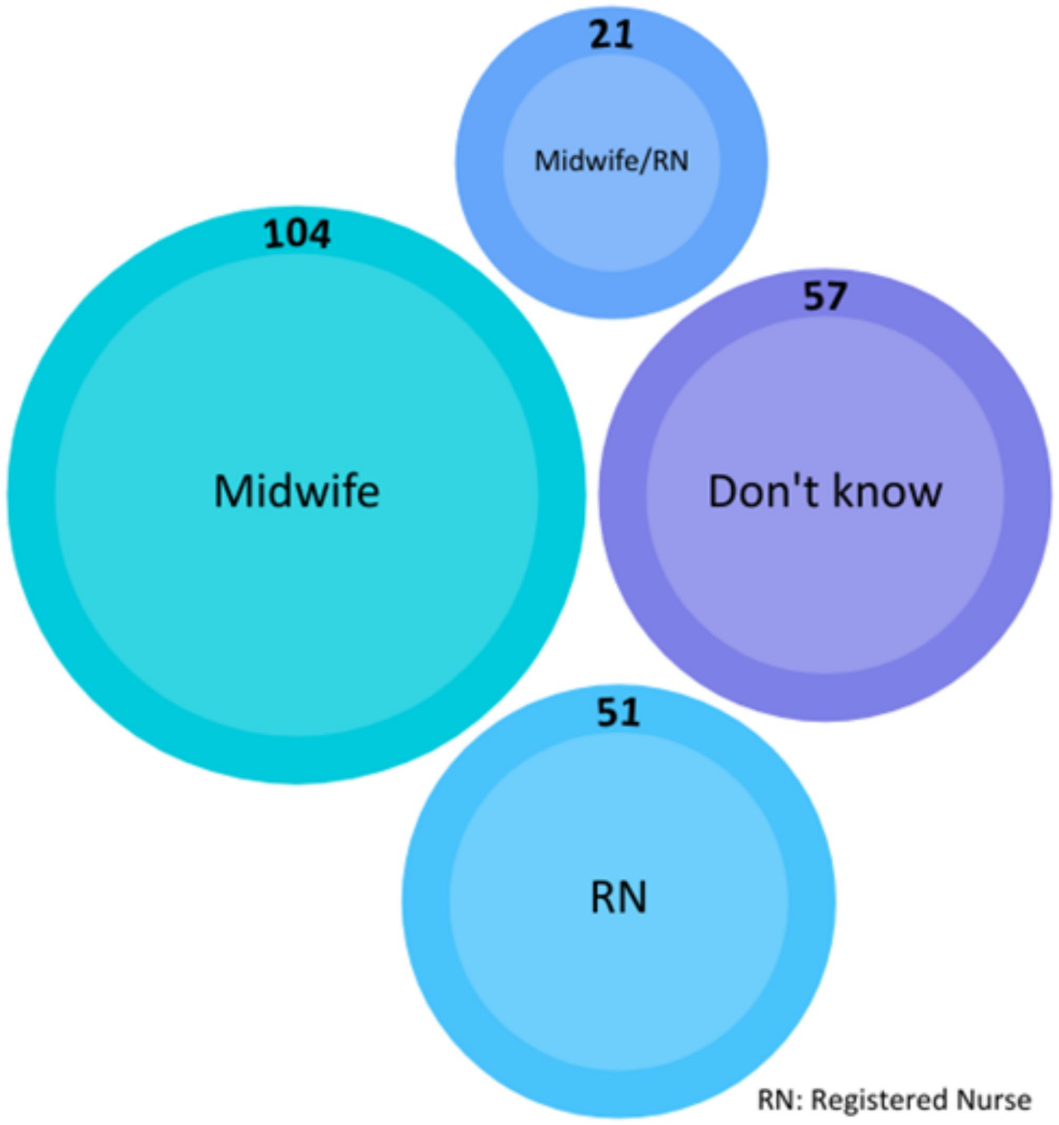
Qualification required to work in acute early pregnancy care by number of responses

In participant workplaces with acute early pregnancy services, being a midwife was the most reported qualification required (104, 59%) (Fig. 3), although nearly one-quarter (24%) of participants did not know what qualification was needed where they worked. Participants’ location impacted the type of service women could access; for example, nearly all participants from South Australia reported that being a midwife was required (27, 93%), compared with those from Western Australia who more commonly reported that being an RN was necessary (31, 47%; χ^2^ p = < 0.001). Most participants from South Australia worked in metropolitan locations (36, 87%) with 25 of these (61%) in a setting with a women’s ED, factors which may impact what qualifications are necessary in individual locations (Table 6).

**Table 6:** Type of acute early pregnancy care service by participant location

Characteristic and participant number (*n*)	General ED *n* (%^^#^)	EPAS model *n* (%^^#^)	Women’s ED *n* (%^^#^)	Maternity Unit/Ward *n* (%^^#^)	Gynae unit *n* (%^^#^)	Women’s Assessment Unit *n* (%^^#^)	
						Maternal/ Fetal	All gestations
Total sample (340)	157 (46.2)	142 (41.8)	83 (24.4)	71 (20.9)	72 (21.2%)	39 (11.2)	44 (12.9)
Metropolitan (239) Rural (101)	88 (36.8) 69 (68.3)	126 (52.7) 16 (15.8)	80 (33.5) 3 (3.0)	47 (19.7) 24 (23.8)	61 (25.5) 11 (10.9)	33 (13.8) 5 (5.0)	41 (17.2) 3 (3.0)
NSW (48) Victoria (61) Queensland (42) South Australia (41) WA (127) Tasmania (4) NT (7) ACT (10)	30 (62.5) 27 (44.3) 20 (47.6) 8 (19.5) 58 (45.7) 3 (75.0) 2 (28.6) 9 (90.0)	26 (54.2) 16 (26.2) 15 (35.7) 25 (61.0) 51 (40.2) 2 (50.0) 1 (14.3) 6 (60.0)	0 (0) 10 (16.4) 3 (7.1) 25 (61.0) 43 (33.9) 0 (0) 1 (14.3) 1 (10.0)	16 (33.3) 11 (18.0) 9 (21.4) 17 (41.5) 11 (8.7) 3 (75.0) 3 (42.9) 1 (10.0)	11 (22.9) 10 (16.4) 8 (19.0) 11 (26.8) 26 (20.5) 2 (50.0) 2 (28.6) 2 (20.0)	10 (20.8) 5 (8.2) 3 (7.1) 7 (17.1) 6 (4.7) 2 (50.0) 0 (0) 5 (50.0)	1 (2.1) 10 (16.4) 3 (7.1) 14 (34.1) 5 (3.9) 3 (75.0) 1 (14.3) 7 (70.0)

**^**Percentages may add up to > 100% as participants could select more than one response; ^#^Valid percentage of respondents in each location; ED: Emergency Department; EPAS: Early Pregnancy Assessment Service; Gynae: Gynaecology; NSW: New South Wales; WA: Western Australia; NT: Northern Territory; ACT: Australian Capital Territory

### Defining midwifery scope in acute early pregnancy care

**Fig. 4:**
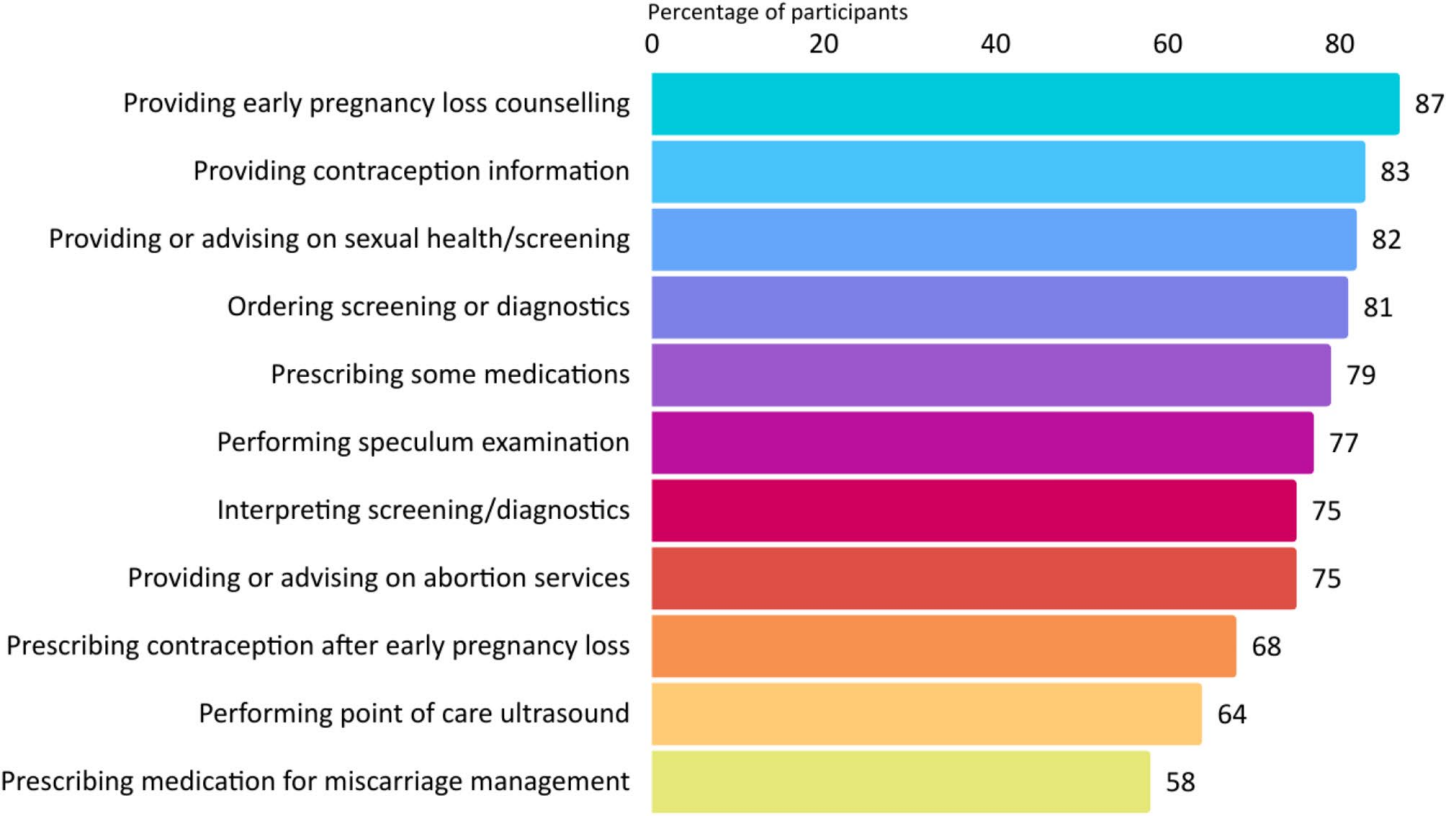
What should midwives’ scope in acute early pregnancy care provision include?

Participants were asked whether specific aspects of acute early pregnancy care provision *should be* part of a midwife’s scope. They frequently identified many items (Fig. 4) including providing counselling for early pregnancy loss (295, 87%) and providing sexual health care (278, 82%). Providing *information* on contraception after early pregnancy loss was also supported by most participants, although significantly less identified that midwives should be *prescribing* this (283, 83% v 230, 68%; χ^2^ p = < 0.001). Three quarters (254, 75%) of participants identified that providing and advising women regarding abortion care should be within the scope of the midwife. The least number of participants identified performing point of care ultrasound (PoCUS) (218, 64%) and prescribing medication for medical management of miscarriage (198, 58%) should be part of midwifery scope.

A higher proportion of midwives selected all scope items in the survey compared to midwifery students. More endorsed midwives identified that midwives’ scope should include all listed items; interpreting screening or diagnostic tests (64, 91% v 162, 72%: χ^2^ p = < 0.001), performing a speculum examination (64, 91% v 166, 74%; χ^2^*p* =.002) or PoCUS (57, 81% v 143, 64%; χ^2^*p* =.006), and prescribing medication for medical management of miscarriage (59, 84% v 121, 54%; χ^2^ p = < 0.001) and contraception after early pregnancy loss (60, 86% v 148, 66%; χ^2^*p* =.002) were significant. A trend was noted regarding midwives who had been registered for more than 10 years, who less frequently selected all scope items compared with less experienced colleagues; provision of abortion options and services was significantly different (101, 86% v 126, 71%; χ^2^*p* =.002). A higher percentage of clinically practising midwives working in a continuity model identified that midwives should prescribe medication for medical management of miscarriage, compared to those in non-continuity models (53, 72% v 81, 57%; χ^2^*p* =.031). This aligns with a similar finding with endorsed midwives and may reflect the higher percentage of endorsed midwives working in continuity models in this study (38; 54% v 36; 12%).

### Potential barriers to midwives providing acute early pregnancy care

Participants selected their ‘top three’ *barriers* to establishing midwives’ role in acute early pregnancy care provision in Australia; these also reflect the barriers most frequently identified by midwife participants (Table 7). A lack of exposure and experience as qualified midwives in acute early pregnancy settings (106, 36%), and a lack of opportunity for midwives to be employed in these services (83, 28%), were most frequently selected. Almost one third of midwives identified women with early pregnancy complications being placed in non-maternity settings was a barrier (87, 30%); this was significant for those midwives with no experience in acute early pregnancy care (37, 41% v 50, 25%; χ^2^*p* =.005). A higher proportion of midwives working in non-metropolitan settings identified the lack of available specialised early pregnancy services in some locations as a barrier (22; 24% v 20; 10%; χ^2^*p* =.002). ‘Other’ barriers reported included resistance from medical staff, lack of recognition and acknowledgement of midwives’ role and scope, and difficulties with government funding arrangements for prescribing. Midwifery students most frequently identified a lack of education (21, 46%), and clinical exposure (23, 50%) as students as potential barriers.

**Table 7:** Barriers to Establishing midwives’ role in acute early pregnancy care in Australia

Potential barriers for midwives	Frequency *n* (%^#^)
Lack of clinical experience and exposure in AEPC settings as qualified midwife	106 (36.1)
Placing women with AEP concerns < 20 weeks in a non-maternity setting	87 (29.6)
Lack of opportunity for midwives to be employed in settings that provide AEPC	83 (28.2)
Uncertainty regarding midwives’ scope in this area of pregnancy care	75 (25.5)
This area of pregnancy care is not prioritised by HSP/my employer	53 (18.0)
This area of pregnancy care is not prioritised by the midwifery profession	52 (17.7)
Lack of clinical exposure and experience in AEPC settings as a midwifery student	52 (17.7)
Lack of education as a midwifery student regarding AEPC	52 (17.7)
Uncertainty regarding whether midwives are responsible for acute care < 20 weeks	52 (17.7)
Staffing or workload constraints e.g., redirection of midwives to women > 20 weeks	52 (17.7)
Lack of individual knowledge or comfort to provide AEPC	45 (15.3)
Specialised AEP services are not available in many locations	42 (14.3)
Other	20 (6.8)

**#**Percentage of midwife sample only; AEPC: Acute early pregnancy care; HSP: Health service provider; AEP: Acute early pregnancy

### Qualitative findings

**Fig. 5:**
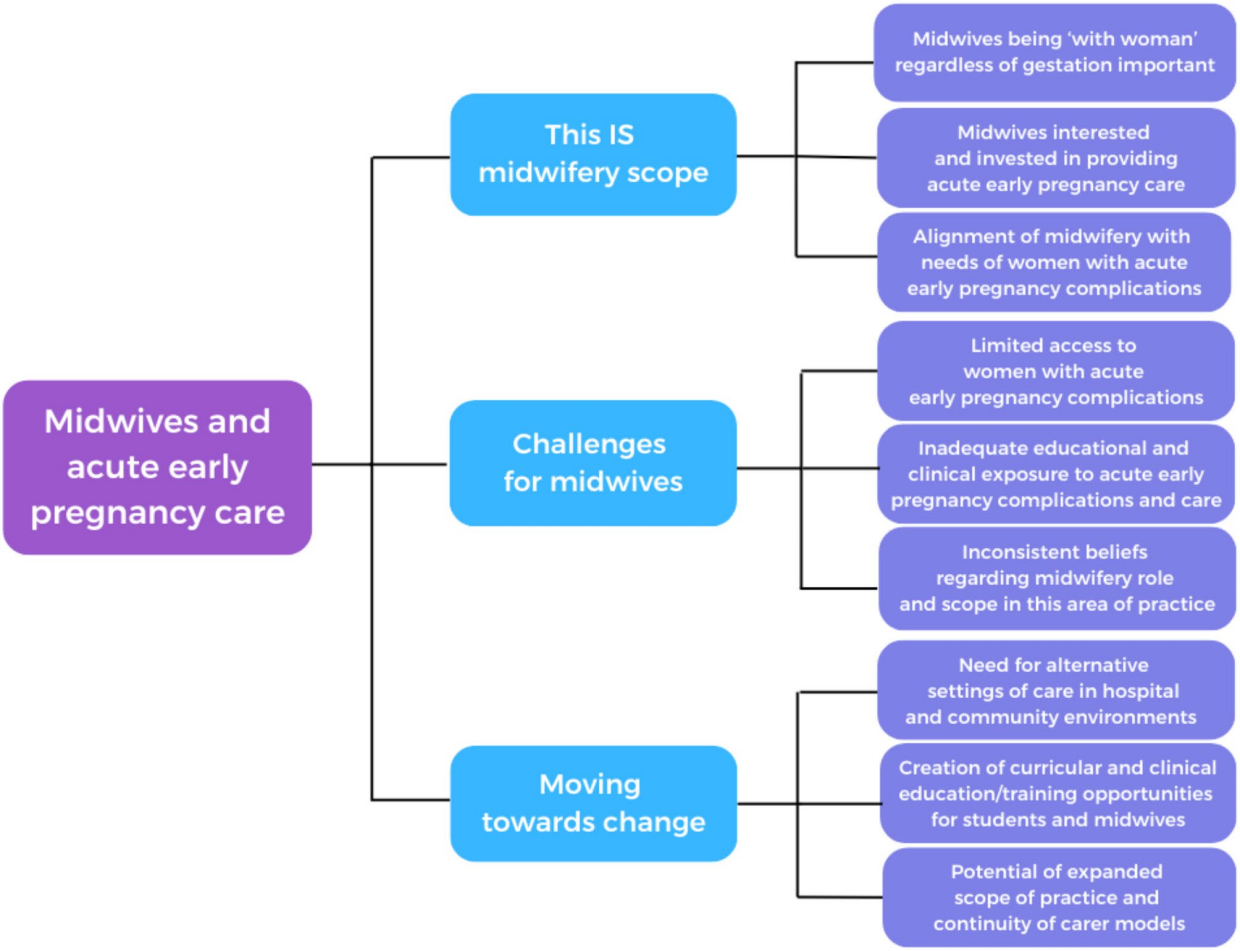
Categorical map of participants comments regarding midwifery role and scope in acute early pregnancy care

Data from free text survey responses were analysed for patterns and summarised into categories [52, 54, 56] (Fig. 5). An exemplar of analysed free-text data is attached (Additional file 3). Participants expressed support for midwives providing care for women with acute early pregnancy complications, identifying both challenges and insightful suggestions to bring midwives and women together at this important time in pregnancy.

### This IS midwifery scope

Participants confirmed that midwifery scope is broad, covering a range of reproductive health care needs that includes the care of pregnant women regardless of gestation or pregnancy outcome. Although a few participants reported RNs can and were providing competent care to women with acute early pregnancy complications, the alignment of midwifery with the expert care of pregnant women was highlighted:

*‘Midwives are experts in women’s reproductive health and should be foundational to any health service that offers early pregnancy assessment…women deserve to be cared for by a midwife throughout the course of their pregnancy*,* no matter the length of their pregnancy or when their need for care may arise’.* P#287.

The philosophical approach of midwifery was identified to be very important to the provision of holistic, woman-centred care for women and families who may be experiencing distress and uncertainty. One midwife described how actively seeking education and training in acute early pregnancy complications in her role as a privately practising midwife (PPM) enabled her to provide care support for the present and future:

*‘When women who engage with me have a loss*,* my role is significant as I can coordinate care and get her the support. I feel that I have made a difference…women have returned to me for care when they fell pregnant again’.* P#189.

Many participants described how they were thinking about acute care in early pregnancy for the first time through completing the survey and were interested in learning more. One student reflected, despite having little exposure to acute early pregnancy complications, of the importance of knowing how to support women at this time:

*‘It is an area however that I am interested in learning more about. I have spoken with women who have experienced early pregnancy loss who have felt unsupported and not sure which services to access for help’*. P#249.

### Challenges for midwives

Participants identified several factors that impacted midwives’ capacity to be ‘with woman’ experiencing unexpected complications in early pregnancy. The structure of acute services in some locations meant that women < 20 weeks were cared for away from maternity settings and midwives. However, participants were mindful that the environment of a traditional maternity unit may not be appropriate for women experiencing early pregnancy loss:

*‘Maternity settings should support a service separated from main maternity but still within maternity for midwives to support women’*. P#187.

Concerns about adequately staffing maternity areas for women at later gestations were also raised. Midwifery workforce shortages may lead to challenges regarding employment priorities:

*‘There is a shortage of midwives to provide continuity of care to women > 20 weeks… if I had to choose*,* I would prefer a midwife cared for the women > 20 weeks as I feel they can make a huge difference’* P#288.

The requirement for RNs to be employed in some acute early pregnancy settings can be particularly problematic for single registration midwives. Even in health services with specialised early pregnancy services that employed midwives, some were excluded from practising if they were not an RN:

*‘I am not allowed to work in early pregnancy care…I have a certificate in sexual and reproductive health and still cannot work in that area because I am not a nurse’* P#37.

Inadequate education and training regarding acute early pregnancy complications and care described by some participants did not align with their beliefs regarding the scope of practice of the modern midwife in Australia. Several midwives also described how a lack of understanding or acknowledgement of a midwife’s scope of practice from other health professionals, or support from health service providers, impacted their ability to work to full scope:

*‘We don’t have policy in our workplace for us [endorsed midwives] to be truly autonomous because they refuse to ‘allow’ us to prescribe. We have to ask RMOs (some with limited understanding) to prescribe meds and order tests*’ P#137.

### Moving towards change

Participants suggested further formal education and clinical training in acute early pregnancy complications and care was necessary for midwives to feel confident to provide this care to women. Although some described learning on the job (e.g., midwives in rural settings), there was a clear desire to provide more significant educational opportunities for students and qualified midwives:

*‘If early pregnancy care is to be included…it needs to be an additional education module post midwifery qual OR the current midwifery training needs to be extended’.* P#237.

The potential for endorsed midwives to have a significant role in acute early pregnancy care provision was suggested by some participants. The capacity for endorsed midwives to prescribe and order diagnostics was seen as an important aspect of scope that should be afforded to all midwives:

*‘Including in the curriculum along with prescribing courses would be hugely beneficial toward enabling midwives to support women and families through full scope of practice’*. P#17.

Even though midwives wanted to provide the most comprehensive care to women with early pregnancy complications, there was also clear support for collaborative practice, and recognition of the benefits of multidisciplinary care for midwives and women:

*‘It can be a big ask for midwives to expand their scope to cover more care. The benefit of collaborative practice allows other professions to be experts in their field (i.e.*,* ultrasound)…allowing midwives to stay woman-centred’.* P#238.

Participants gave insightful suggestions regarding how the midwifery profession can take positive steps to improve how acute early pregnancy care is structured, and to facilitate increased midwifery access for women experiencing complications. Early engagement of women with midwives in a continuity model would provide women with support, particularly if they go on to experience a complication such as miscarriage. Several midwives suggested much of the care women with early pregnancy complications need can be provided by midwives, potentially in out-of-hospital settings. If community care is not possible, referring women to a maternity service ensures ongoing access to midwives:

*‘Our MGP has a pilot program…we accept early referrals for women experiencing hyperemesis gravidarum so they can present to the maternity unit for IV anti-emetics*,* rehydration*,* get scripts etc. rather than presenting to the ED’* P#227.

Alternatives need to be considered to ensure midwifery care can also be provided away from other pregnant women and babies. In addition to considering how hospital early pregnancy services are structured and located, one community-based midwife described how she can and could provide care in the instance of pregnancy loss without women needing to attend a maternity setting:

*‘I have had exposure to supporting women following miscarriage and wanting expectant management…have always wondered if I could manage medical [treatment] in a community setting as an endorsed midwife’* P#307.

## Discussion

This study provides a novel insight into the perspectives of midwives and midwifery students regarding acute early pregnancy care provision in Australia. Study participants report a strong interest in providing care for women and families experiencing acute early pregnancy complications and confirm that midwives, both as autonomous practitioners and in collaboration with medical and allied health colleagues, are best placed to provide this care.

### Acknowledging and clarifying midwifery scope in acute early pregnancy care

Participants in this study recognised that midwives could provide a wide range of physical and psychosocial care for women with acute complications in early pregnancy, as well as provide acute reproductive care to women who are not pregnant. Participants reported while both midwives and RNs provide acute care in early pregnancy, in some settings being an RN was obligatory. These findings are consistent with other literature describing acute early pregnancy care settings and staffing in Australia [14, 16, 44, 57]. Whilst this research did not seek to question the professional capabilities of nurses, midwives have the defined scope to provide reproductive healthcare to women that includes care before, during and after pregnancy [2, 3]. With nearly half the participants in this study *not* RNs, a finding that reflects the upward trend in single registration midwives in Australia [39, 41, 58], equity of access for all midwives to employment opportunities in acute early pregnancy and reproductive health care settings should be a clear objective for health service providers.

While midwifery practice has clear regulatory guidance in Australia and internationally [2–6], each midwives’ scope is also influenced by individual competence and comfort, policies and expectations of employers, and the needs of women presenting for care [59]. Although participants in this study were supportive of midwives caring for women with a range of acute early pregnancy needs, some suggested additional qualifications might be required. Whilst extended scope activities such as prescribing and ordering diagnostic tests require an endorsement qualification in Australia [42, 59], the potential for midwives to provide comprehensive care for women with acute early pregnancy complications is not dependent on this endorsement. Appropriately educated, skilled and supported midwives [59] can counsel women about management options for miscarriage, provide contraception information, perform an ultrasound or a conduct a speculum examination [16]. The value of emotional support and psychosocial care, skills that study participants identified to be important and that all midwives are ideally placed to provide [3, 9], are highly valued by women and families [26]. The strong response from endorsed midwives in this study did however provide valuable insight into extended midwifery scope in acute early pregnancy care, highlighting the potential of endorsed midwives to provide holistic care in community and hospital settings. However, barriers remain in their capacity to utilise extended skills, with a lack of authority to perform tasks such as prescribing at a local service level reported in this study. This needs to be addressed across all aspects of midwifery practice at a local and national level [59] as the number of midwives with endorsement increases [41, 58].

Fewer participants expressed support for midwives performing skills traditionally the responsibility of other health professionals, including early pregnancy ultrasound and prescribing medication for miscarriage. Participants reported that working in a multidisciplinary team enabled midwives to focus on supporting and advocating for the woman and her family, central components of midwifery care [9]. Some did express concern however that other health professionals, particularly doctors, did not understand, support or ‘allow’ a midwife to fulfil their scope of practice. Study findings regarding some midwives’ reporting a lack of workplace scope of practice guidance for both midwives and other health professionals suggest more clarity is needed in this area. Interdisciplinary education and training regarding acute early pregnancy complications and care may assist midwives and doctors to better understand the unique role and scope of each profession. Facilitating respectful multidisciplinary collaboration may support scope fulfilment across disciplines, leading to more co-operative clinical practice and improved care for women and families [60].

### Education, knowledge and confidence

Many participants received limited education about acute early pregnancy complications as student midwives, and even less reported having clinical placements in acute early pregnancy care settings. This aligns with qualitative findings from earlier research [16] and highlights the need for a review of the curricular content of midwifery programs and the clinical exposure of students to women with acute early pregnancy complications. Despite limited student exposure, most qualified midwives reported fair to strong knowledge of miscarriage, HG and ectopic, perhaps a reflection for some of their exposure to these topics during their midwifery education. Less reported strong knowledge of how to provide the care for women with these conditions, suggesting a lack of integration between theoretical learning and clinical practice consolidation, a concept not new to health professional education, including midwifery [61]. Midwives should and must receive both theoretical education *and* clinical opportunities as students that align with scope of practice guidance for the modern midwife [2, 3, 59]. With midwifery scope, both in Australia and internationally, continuing to evolve [2, 42, 62], it is timely to consider whether current education programs are preparing midwives to practice both autonomously and collaboratively throughout a woman’s reproductive years.

With a higher percentage of midwives with acute early pregnancy experience reporting to have had clinical placements as midwifery students, consolidating theoretical learnings with practical placements may improve midwives’ confidence to work in acute early pregnancy settings once qualified. Consideration should be given in the next review of accreditation standards for midwifery education programs in Australia [63] to the inclusion of more comprehensive language regarding the breadth of midwifery scope in the antenatal period, and early engagement in continuity of care experiences that maximise student’s opportunities to be with women under 20 weeks gestation. A more diverse range of community and hospital-based clinical placement options [64] that further expose students to women who experience acute complications in early pregnancy should also be explored.

### Enabling midwives to provide acute care in early pregnancy

Participants agreed that women experiencing complications such as miscarriage or HG *should* be cared for by midwives in a maternity setting, however they were less certain regarding the appropriateness of this environment for women experiencing early pregnancy loss, a finding supported in other literature [16, 65]. Most participants agreed this group of women *should* and *want* to be separated from other women and babies, although they also want women to be with midwives whenever possible. It is important to acknowledge the juxtaposition of these two findings and work towards solutions that achieve two goals – midwives providing care for women with acute complications in early pregnancy, and women receiving midwifery care in a setting they feel safe and comfortable.

Nearly half of participants reported women were cared for in a general ED where they worked, and there is some evidence of midwives providing acute early pregnancy education, staff support and clinical care in general ED settings in Australia [16, 66]. Whilst some women with acute early pregnancy complications require urgent hospital treatment [31, 67], most experiencing symptoms such as bleeding who present for emergency care do not require urgent intervention [67]. Midwives in this study described providing care for women in midwife-led EPAS settings, group practice and collaborative hybrid home/hospital care models, providing women with support from midwives, not just for early pregnancy loss but also for challenging conditions such as HG [30]. Pilot programs exist in Australia that seek to provide women with acute early pregnancy concerns telehealth access to midwives [57]. The feasibility of community based EPAS models, which enable collaborative care to be provided away from a hospital maternity setting [65], and midwife-led EPAS models [18] should also be explored. Consultation with women, midwives, maternity care consumers, and health service providers should form part of the upcoming nationwide audit of early pregnancy assessment models commissioned by the Australian Government in 2024 [68].

The most appropriate setting for care of women acutely unwell is dependent on the location of the woman, the accessibility of health care, and the availability of appropriate health care professionals [69]. For clinically stable women, EPAS models staffed by midwives, and other expert pregnancy clinicians (e.g., doctors, sonographers) when required, should be considered for initial and ongoing care. Specialist women’s early pregnancy services, including women’s ED and EPAS models, were more commonly reported by participants working in metropolitan settings. Strategies to provide women presenting to general ED settings in rural and remote locations access to midwives and other specialised pregnancy clinicians requires further exploration through a consultative process with local stakeholders. The role metropolitan-based services such as the midwife-led South Australian Virtual Women’s Assessment Service [57] may have in supporting midwives to provide primary care to women in their own communities is also worthy of further investigation.

The recently published recommendations from the first national midwifery workforce review in Australia [39] provide an important scaffold on which to begin addressing midwifery service provision across a range of settings and models of care. While midwives’ role preference in the workforce survey was well distributed, those working in continuity models were less likely to report wanting to leave midwifery [39]. Midwives in this study who worked in continuity models reported prior or current acute early pregnancy exposure more frequently than those working in non-continuity models. With women in Australia expressing a strong desire for consistent, personalised, timely and accessible maternity care with known providers [70], the capacity for midwives in both shift-based and continuity models to provide this for women with acute complications in early pregnancy needs to be explored.

### Strengths and limitations

To our knowledge, this is the first study exploring midwives and midwifery students’ practice in acute early pregnancy care provision in Australia. The study sample demonstrated variation in midwives’ age, qualifications, educational background, geographical location and work environment. The inclusion of midwifery students provided valuable insight into current educational and clinical preparation of midwives to provide care for women with acute early pregnancy complications. Whilst recruitment strategies were national, Tasmania, the Northern Territory and the Australian Capital Territory had less participants, reflecting the smaller populations in these locations.

Although the study focus was midwifery practice in acute early pregnancy care provision, the wider role and scope of midwives in the provision of other aspects of sexual and reproductive healthcare was also explored. This provides important benchmarking data on which to build future research regarding the role and scope of practice of the modern midwife in Australia, including the care of women who are not pregnant.

This type of exploratory study does not seek to provide a sample population that is representative of the entire study population, or to make generalisations about the study results in relation to the study population. However, given the diversity of the characteristics of the study sample, results may be applicable and useful in selected local and international contexts. Although recruitment specified acute early pregnancy experience was not required, selection bias may have occurred in terms of midwives with acute early pregnancy care knowledge or experience being more likely to engage in the study. Recall bias may have impacted responses regarding pre-registration midwifery education and training exposure to acute early pregnancy care, particularly for those participants who had been registered for some time.

## Conclusions

Participants in this study have clearly expressed support for midwives providing acute care to women experiencing complications in early pregnancy, but do not necessarily feel prepared or professionally enabled to do so. As the scope of the midwife in Australia and internationally includes the care of women across the reproductive spectrum, adequate preparation of midwives to provide acute early pregnancy care is vital. Findings have utility in supporting policy, education and service review and highlight further gaps in evidence for future research. The additional scope of the endorsed midwife in this area of practice requires further exploration. Consideration of the current curricular and clinical placement requirements for midwifery students in Australia is necessary, alongside collaborative engagement of consumers, midwives, pregnancy clinicians and health service providers, to enact organisational change that supports and facilitates midwives to be with women experiencing acute complications at any gestation of pregnancy.

## Electronic supplementary material

Below is the link to the electronic supplementary material.

Supplementary Material 1 Additional file 1: Participant survey: Questions for both participant cohorts, layout order and online formatting and logic commands

Supplementary Material 2 Additional file 2: Full table: Participant’s knowledge of acute early pregnancy complications and care – descriptive statistics and associations (binary responses) between sub-groups

Supplementary Material 3 Additional file 3: Qualitative analysis exemplar: Process and application to participant’s comment

## Data Availability

Data is provided within the manuscript or supplementary information files.

## Abbreviations

ED: Emergency Department
RN: Registered Nurse
EPAS: Early Pregnancy Assessment Service
NMBA: Nursing and Midwifery Board of Australia
HG: Hyperemesis Gravidarum
b-hCG: Beta human chorionic gonadotrophin
PoCUS: Point-of-Care Ultrasound
PPM: Privately Practising Midwife
RMO: Resident Medical Officer
MGP: Midwifery Group Practice
IV: Intravenous
