# Supplementary material for: Exploring midwifery role and scope in acute early pregnancy care: a survey of midwives and midwifery students in Australia
Source: BMC Pregnancy Childbirth. 2025 Apr 16;25:458. doi: 10.1186/s12884-025-07567-3 (PMC12004735; doi:10.1186/s12884-025-07567-3)
Supplement: Supplementary file 1 — Supplementary Material 1 Additional file 1: Participant survey: Questions for both participant cohorts, layout order and online formatting and logic commands [file 12884_2025_7567_MOESM1_ESM.docx]

**Additional file 1 – Participant survey**

**Block 1**

Qualtrics splash page [Text/Graphic]

**Thank you** for your interest in this survey, which aims to explore the role and scope of practice of midwives in acute early pregnancy care provision in Australia. A full participant information form is available here [link].

*Is this survey for me?*

This survey is for **any midwife currently registered with the NMBA, or any current midwifery student in Australia**, regardless of your experience or knowledge of acute early pregnancy care.

*What do you mean by acute early pregnancy care?*

- **Unscheduled care** under 20 weeks (e.g., in an emergency department or other urgent care service)
- Includes **‘sub-acute’** care provided in settings such as acute gynaecological or early pregnancy assessment service (EPAS) models

It **does not include**:

- Acute presentations under 20 weeks that are **not pregnancy related** (e.g., trauma)
- Scheduled (routine) antenatal care or maternal-fetal medicine care appointments

*Consent and commence the survey*

I meet the inclusion criteria, have read and understood the participant information form, and consent to participate in this survey – **Click** [hurdle question to consent and enter the survey]

**Demographics** [Multiple choice format]

Social demographics

**Q1***: Age* (years)

1. 18-24
2. 25-34
3. 35-44
4. 45-54
5. 55-64
6. 65+

**Q2:** *Gender*

1. Woman or female
2. Man or male
3. Non-binary
4. A different term (please specify)
5. Prefer not to answer

**Q3:** *Are you of Aboriginal and/or Torres Strait Islander origin*?

1. No
2. Yes, Aboriginal
3. Yes, Torres Strait Islander
4. Yes, both Aboriginal and Torres Strait Islander

**Q4:** *Do you speak a language other than English at home?*

1. No
2. Yes (please specify)

**Q5:** *State or Territory of usual practice*

1. New South Wales
2. Victoria
3. Queensland
4. South Australia
5. Western Australia
6. Tasmania
7. Northern Territory
8. Australian Capital Territory
9. Other Territories

**Q6:** *Location of practice:*

1. Metropolitan
2. Larger Regional
3. Smaller Rural
4. Remote Community

**Q ‘13’:** Professional qualifications (select ALL that apply):

1. Midwife
2. Registered Nurse
3. Endorsed Midwife
4. IBCLC Lactation Consultant
5. Maternal Child Health/Child Health
6. Other e.g., paramedic, teacher (please specify)
7. Nil

**Q7:** Current professional status in Australia: [cohort split here]

1. I am currently registered as a midwife
2. I am currently a midwifery student

| Cohort 1 - Midwife | Cohort 2 – Midwifery student |
| --- | --- |
| **Q8:** Qualification completed for *initial registration* *as a midwife*: (Select ONE)   1. Hospital-based Certificate in Midwifery 2. Undergraduate Degree in Midwifery 3. Dual Degree in Midwifery and Nursing 4. Postgraduate Diploma in Midwifery 5. Postgraduate Masters in Midwifery   **Q9:** Highest post-secondary education attained: (Select ONE)   1. Hospital based diploma or certificate 2. Postgraduate Diploma 3. Bachelor’s Degree 4. Masters 5. PhD   [option to free text discipline e.g., nursing]  **Q10:** How long have you been registered as a midwife in Australia?   1. 5 years or less 2. 6-10 years 3. 11-15 years 4. 16-20 years 5. 21+ years   **Q11:** In your *primary or main job*, what sector are you employed in? (Select ONE)   1. Public sector health service 2. Private sector health service 3. Private practice 4. Other – please specify   **Q12:** *Primary or main* employment model of care: (Select ONE)   1. Continuity model e.g., Midwifery Group Practice, Birth Centre, Privately Practising Midwife 2. Core/non-continuity/ non-rotational model e.g., Birth Suite, Outpatient clinics, Postnatal Ward, Emergency Department, Early Pregnancy Assessment, Antenatal Assessment, Theatre, Home Visiting 3. Rotational hospital - including graduate program – moving between various areas 4. Education, Research or Academia 5. Management or Executive 6. Other – please specify | **Q14:** Qualification being undertaken for *initial registration* as a midwife: (Select ONE)   1. Undergraduate Degree in Midwifery 2. Dual Degree in Midwifery and Nursing 3. Postgraduate Diploma of Midwifery 4. Postgraduate Masters of Midwifery   **Q15:** Highest post-secondary education attained **prior** to coming to midwifery: (Select ONE)   1. None 2. TAFE diploma or certificate 3. Hospital based diploma or certificate 4. Postgraduate Diploma 5. Bachelor’s Degree 6. Masters 7. PhD   [option to free text discipline e.g., nursing] |

**Block 2**

**Education and exposure to acute early pregnancy care in Australia** [Multiple choice]

The following statements relate to your *education and exposure* to acute early pregnancy

complications and care in Australia.

| Midwife | Midwifery student |
| --- | --- |
| **Q16:** I *learnt* about acute early pregnancy complications and care as a *midwifery student*   1. Yes 2. No 3. Unsure | **Q20:** I *am learning* about acute early pregnancy complications and care as a midwifery student   1. Yes 2. No 3. Unsure 4. Not yet |
| **Q17:** I *had* clinical placements in acute early pregnancy care settings as a *midwifery student*   1. Yes 2. No 3. Unsure | **Q21:** I *have* clinical placements in acute early pregnancy care settings as a midwifery student   1. Yes 2. No 3. Unsure |
| **Q18:** What is your *clinical experience* regarding the provision of acute early pregnancy care **as a midwife** in Australia? (Select ONE)   1. I have never provided acute early pregnancy care 2. I have previously provided acute early pregnancy care but don’t anymore 3. I am currently providing acute early pregnancy care   [Skip logic] Response 1 – **skip** Q19 and go to Q22; Response 2 – go to Q19 **and** Q22; Response 3 – go to Q19 but **skip** Q22 |  |

**Q19:** What enabled or motivated you to work in acute early pregnancy care (past or present)? Select ALL that apply

1. I had exposure to acute early pregnancy care as a student or as a graduate
2. I worked in an acute early pregnancy setting as a nurse
3. My exposure was opportunistic e.g., through antenatal clinic, relieving in a role, merging of early and later pregnancy assessment services
4. I work/have worked in an emergency department setting where women present in early pregnancy
5. I have worked in a gynaecology setting
6. I have worked in sexual and reproductive health
7. I actively sought out a role as a midwife in an acute early pregnancy care setting
8. I have had personal experience with acute early pregnancy complications and/or care
9. I was attracted to the working hours of the role
10. Other (please specify) __________________________________________________

**Q22:** If it was possible and relevant to your current setting, would you be interested in providing acute early pregnancy care as a midwife?

1. Yes
2. No
3. Unsure

**Block 3**

**Knowledge and confidence regarding acute early pregnancy complications and care** [Matrix table]

*Knowledge*

**Q23:** How would you rate your *overall* knowledge of the following acute early pregnancy *complications*?

Miscarriage:

1. Strong
2. Fair
3. Limited
4. None

Ectopic pregnancy:

1. Strong
2. Fair
3. Limited
4. None

Hyperemesis gravidarum:

1. Strong
2. Fair
3. Limited
4. None

**Q24:** How would you rate your *overall* knowledge of the *care* (i.e., assessment, treatment, and management) of the following acute early pregnancy complications?

Miscarriage:

1. Strong
2. Fair
3. Limited
4. None

Ectopic pregnancy:

1. Strong
2. Fair
3. Limited
4. None

Hyperemesis gravidarum:

1. Strong
2. Fair
3. Limited
4. None

*Confidence in clinical practice*

**Q25:** How would you rate your *level of confidence* to provide acute early pregnancy care in the following situations? [Matrix table]

[Physical care]

Complete an assessment, and organise analgesia, for a woman experiencing acute pain <20 weeks

1. Strongly confident
2. Fairly confident
3. Limited confidence
4. Not confident

Perform a speculum examination on a woman with vaginal bleeding <20 weeks

1. Strongly confident
2. Fairly confident
3. Limited confidence
4. Not confident

Recognise and respond to clinical deterioration in a woman with heavy vaginal bleeding <20 weeks

1. Strongly confident
2. Fairly confident
3. Limited confidence
4. Not confident

Administer methotrexate for ectopic pregnancy

1. Strongly confident
2. Fairly confident
3. Limited confidence
4. Not confident

Identify an acceptable upward trend in quantitative beta human chorionic gonadotrophin (beta-hCG) levels in a woman at 6 weeks of pregnancy

1. Strongly confident
2. Fairly confident
3. Limited confidence
4. Not confident

Manage the third stage of labour for a woman who has miscarried under 20 weeks gestation

1. Strongly confident
2. Fairly confident
3. Limited confidence
4. Not confident

*[Psychosocial support and education]*

Provide support and education for a woman with a diagnosed pregnancy loss <20 weeks (e.g., miscarriage)

1. Strongly confident
2. Fairly confident
3. Limited confidence
4. Not confident

Explain to a woman the treatment options and post operative support for a diagnosed tubal ectopic pregnancy

1. Strongly confident
2. Fairly confident
3. Limited confidence
4. Not confident

Provide information to a woman/partner regarding the community resources available that offer support and information following early pregnancy loss

1. Strongly confident
2. Fairly confident
3. Limited confidence
4. Not confident

Identify when a woman with an acute early pregnancy complication should be offered referral to psychological or social care services

1. Strongly confident
2. Fairly confident
3. Limited confidence
4. Not confident

Explain to a junior doctor the available treatment options for a diagnosed first trimester missed miscarriage

1. Strongly confident
2. Fairly confident
3. Limited confidence
4. Not confident

**Block 4**

**Scope of practice; Setting of care** [Matrix table]

*Scope of practice*

**Q26 (Midwife) and Q27 (Student):** Please indicate your *level of agreement* with the following statements:

Midwives’ scope of practice in Australia includes the care of women with **acute complications** under 20 weeks gestation

1. Strongly agree
2. Agree
3. Disagree
4. Strongly disagree

Midwives’ scope of practice in Australia includes the care of women experiencing **pregnancy loss** under 20 weeks gestation

1. Strongly agree
2. Agree
3. Disagree
4. Strongly disagree

Midwives practicing in Australia should provide care to **non-pregnant women** with acute reproductive health concerns e.g., heavy menstrual bleeding, endometriosis, acute pain following an IUD insertion

1. Strongly agree
2. Agree
3. Disagree
4. Strongly disagree

Where I currently work, there are clinical guidelines or policies that clearly outline what midwives can and cannot do as part of their role [Midwife only]

1. Strongly agree
2. Agree
3. Disagree
4. Strongly disagree

Where I currently work, there are guidelines or policies that clearly outline what **my colleagues** (e.g., doctors, RNs, other midwives) can and cannot do as part of their role [Midwife only]

1. Strongly agree
2. Agree
3. Disagree
4. Strongly disagree

Midwives are the best qualified professionals to provide acute care for pregnant women under 20 weeks

1. Strongly agree
2. Agree
3. Disagree
4. Strongly disagree

Registered nurses who are **not** midwives have the professional and educational preparation to provide acute early pregnancy care

1. Strongly agree
2. Agree
3. Disagree
4. Strongly disagree

A woman experiencing a threatened miscarriage at 7 weeks **should be** cared for in a maternity setting rather than an emergency or gynaecology setting

1. Strongly agree
2. Agree
3. Disagree
4. Strongly disagree

Women requiring acute treatment for hyperemesis gravidarum under 20 weeks **should be** cared for in a maternity setting rather than an emergency or gynaecology setting

1. Strongly agree
2. Agree
3. Disagree
4. Strongly disagree

Women and families experiencing early pregnancy loss **want to be** cared for separate from other pregnant and postnatal women

1. Strongly agree
2. Agree
3. Disagree
4. Strongly disagree

Women and families experiencing early pregnancy loss **should be** cared for separate from other pregnant and postnatal women

1. Strongly agree
2. Agree
3. Disagree
4. Strongly disagree

Women recovering from surgery for an ectopic pregnancy **should be** cared for in a gynaecology or surgical setting rather than a maternity setting

1. Strongly agree
2. Agree
3. Disagree
4. Strongly disagree

**Block 5**

**Final questions**

**Q28:** To provide acute early pregnancy care to women under 20 weeks gestation in my setting, you **must** be: (select only ONE)

1. A midwife
2. A registered nurse
3. A dual qualified midwife *and* a registered nurse
4. I don’t know
5. We do not have acute early pregnancy care services where I work
6. I don’t currently have a clinical setting

**Q29:** Where are women with acute early pregnancy complications under 20 weeks cared for in your setting? (Select ALL that apply)

1. General Hospital Emergency Department
2. Women’s Hospital Emergency Department
3. Early Pregnancy Assessment Service/Unit/Clinic (EPAS/EPAU/EPAC)
4. Pregnancy Assessment Unit (i.e., acute presentations at all gestations)
5. Maternal/Fetal Assessment Unit/Day Assessment
6. Maternity Unit, Ward or Obstetric Service
7. Acute Gynaecology Service, Clinic or Ward
8. My setting does not provide services for acute early pregnancy care
9. I don’t currently have a clinical setting
10. Other (please specify) _____________________________

**Q30:** Midwives’ scope of practice in *acute early pregnancy care* provision **should** include (Select ALL that apply):

1. Ordering screening or diagnostic tests e.g., bloods, ultrasound
2. Interpreting screening or diagnostic tests
3. Providing counselling for women with acute early pregnancy complications or loss
4. Performing a speculum examination
5. Prescribing medication for medical management of miscarriage (e.g., mifepristone/misoprostol)
6. Performing point-of-care ultrasound in an early pregnancy population
7. Prescribing some medications in an acute early pregnancy population (e.g., analgesia, Anti-D, anti-emetic)
8. Providing contraception information for postpartum women following early pregnancy loss
9. Prescribing contraception for postpartum women following early pregnancy loss
10. Providing or advising on sexual health/screening
11. Providing or advising on abortion options and services

**Q31:** What are the **top three barriers** to establishing midwives’ role in acute early pregnancy care in Australia? (Allow to select UP TO THREE)

1. Lack of education *as a student* regarding acute early pregnancy care
2. Lack of clinical exposure and experience in acute early pregnancy care settings *as a student*
3. Lack of clinical exposure and experience in acute early pregnancy care settings *as a qualified midwife*
4. Uncertainty regarding whether midwives are responsible for acute care under 20 weeks gestation
5. Uncertainty regarding midwives’ scope of practice in this area of pregnancy care
6. Lack of opportunity for midwives to be employed in settings that provide acute early pregnancy care
7. Placing women with acute concerns under 20 weeks in a non-maternity setting
8. Lack of individual knowledge or comfort to provide acute early pregnancy care
9. Specialised acute early pregnancy services are not available in many locations
10. This area of pregnancy care is not prioritised by the midwifery profession
11. This area of pregnancy care is not prioritised by health service providers/my employer
12. Staffing or workload constraints – e.g., redirection of midwives into areas with women over 20 weeks
13. Other – please specify

**Block 6**

**Free text response ‘wrap up’ question**

**Q32:** Do you have any further comments about midwives’ role and scope of practice in acute early pregnancy care in Australia? ________________________________________________________________________________________________________________________________________________________________________________________________________________________________________________________________________________________________________________________________________

END of survey

THANKYOU once again for your time completing this survey. Your responses have been recorded.
