# Supplementary material for: Exploring midwifery role and scope in acute early pregnancy care: a survey of midwives and midwifery students in Australia
Source: BMC Pregnancy Childbirth. 2025 Apr 16;25:458. doi: 10.1186/s12884-025-07567-3 (PMC12004735; doi:10.1186/s12884-025-07567-3)
Supplement: Supplementary file 2 — Supplementary Material 2 Additional file 2: Full table: Participant’s knowledge of acute early pregnancy complications and care – descriptive statistics and associations (binary responses) between sub-groups [file 12884_2025_7567_MOESM2_ESM.docx]

**Additional file 2**: Participants knowledge of acute early pregnancy complications and care – descriptive statistics and associations (binary response format) between sub-groups

*Knowledge - Condition itself*

| Miscarriage - All participants | Strong or fair knowledge  n (%^) | Limited or no knowledge  n (%^) | p-value |
| --- | --- | --- | --- |
| Whole sample (n^^=327) | 272 (83.2%) | 55 (16.8%) | - |
| Professional group  Midwives (n=287)  Midwifery students (n=40) | 246 (85.7%)  26 (65.0%) | 41 (14.3%)  14 (35.0%) | **p=.001*** |
| Location of practice^+^  Metropolitan (n=229)  Rural or remote (n=98) | 190 (83.0%)  82 (83.7%) | 39 (17.0%)  16 (16.3%) | p=.876 |
| Professional qualifications  Registered Nurse (RN) (n=173)  Not an RN (n=154) | 147 (85.0%)  125 (81.2%) | 26 (15.0%)  29 (18.8%) | p=.359 |
| Miscarriage – Midwives only | **Strong or fair knowledge**  **n (%^)** | **Limited or no knowledge**  **n (%^)** | **p-value** |
| Professional qualifications  Midwife and RN (n^^=164)  Midwife but not RN (n^^=123)  Endorsed midwife (n=70)  Midwife - not endorsed (n=217) | 140 (85.4%)  106 (86.2%)  58 (82.9%)  188 (86.6%) | 24 (14.6%)  17 (13.8%)  12 (17.1%)  29 (14.3%) | p=.846  p=.432 |
| Years of registration^+^  10 or less (n=114)  Greater than 10 years (n=173) | 92 (80.7%)  154 (89.0%) | 22 (19.3%)  19 (11.0%) | **p<.049*** |
| Primary model of care^+^  Continuity (n=71)  Non-continuity/Rotational (n=142) | 62 (87.3%)  117 (82.4%) | 9 (12.7%)  25 (17.6%) | p=.354 |
| Experience in acute early pregnancy care^+^  None (n=89)  Prior or current (n=198) | 65 (73.0%)  181 (91.4%) | 24 (27.0%)  17 (8.6%) | **p=<.001*** |
| Ectopic pregnancy – all participants | **Strong or fair knowledge**  **n (%^)** | **Limited or no knowledge**  **n (%^)** | **p-value** |
| Whole sample n^^=327 | 222 (67.9%) | 105 (32.1%) | - |
| Professional group  Midwives (n=287)  Midwifery students (n=40) | 204 (71.1%)  18 (45.0%) | 83 (28.9%)  22 (55.0%) | **p=<.001*** |
| Location of practice^+^  Metropolitan (n=229)  Rural or remote (n=98) | 155 (67.7%)  67 (68.4%) | 74 (32.3%)  31 (31.6%) | p=.904 |
| Professional qualifications  Registered Nurse (n=173)  Not an RN (n=154) | 130 (75.1%)  92 (59.7%) | 43 (24.9%)  62 (40.3%) | **p=.003*** |
| Ectopic pregnancy – Midwives only | **Strong or fair knowledge**  **n (%^)** | **Limited or no knowledge**  **n (%^)** | **p-value** |
| Professional qualifications  Midwife and RN (n=164)  Midwife but not an RN (n=123)  Endorsed midwife (n=70)  Midwife but not endorsed (n=217) | 125 (76.2%)  79 (64.2%)  51 (72.9%)  153 (70.5%) | 39 (23.8%)  44 (35.8%)  19 (27.1%)  64 (29.5%) | **p=.027***  p=.706 |
| Years of registration^+^  10 or less (n=114)  Greater than 10 years (n=173) | 65 (57.0%)  139 (80.3%) | 49 (43.0%)  34 (19.7%) | **p=<.001*** |
| Primary model of care^+^  Continuity (n=71)  Non-continuity/Rotational (n=142) | 44 (62.0%)  102 (71.8%) | 27 (38.0%)  40 (28.2%) | p= .144 |
| Experience in acute early pregnancy care^+^  None (n=89)  Prior or current (n=198) | 43 (48.3%)  161 (81.3%) | 46 (51.7%)  37 (18.7%) | **p=<.001*** |
| Hyperemesis gravidarum – all participants | **Strong or fair knowledge**  **n (%^)** | **Limited or no knowledge**  **n (%^)** | **p-value** |
| Whole sample n^^=327 | 277 (84.7%) | 50 (15.3%) | - |
| Professional group  Midwives (n=287)  Midwifery students (n=40) | 251 (87.5%)  26 (65.0%) | 36 (12.5%)  14 (35.0%) | **p=<.001*** |
| Location of practice^+^  Metropolitan (n=229)  Rural or remote (n=98) | 197 (86.0%)  80 (81.6%) | 32 (14.0%)  18 (18.4%) | p=.312 |
| Professional qualifications  Registered Nurse (n=173)  Not an RN (n=154) | 149 (86.1%)  128 (83.1%) | 24 (13.9%)  26 (16.9%) | p=.450 |
| Hyperemesis gravidarum - Midwives only | **Strong knowledge**  **n (%^)** | **Limited knowledge**  **n (%^)** | **p-value** |
| Professional qualifications  Midwife and RN (n=164)  Midwife but not an RN (n=123)  Endorsed midwife (n=70)  Midwife but not endorsed (n=217) | 143 (87.2%)  108 (87.8%)  61 (87.1%)  190 (87.6%) | 21 (12.8%)  15 (12.2%)  9 (12.9%)  27 (12.4%) | p=.877  p=.927 |
| Years of registration^+^  10 or less (n=114)  Greater than 10 years (n=173) | 95 (83.3%)  156 (90.2%) | 19 (16.7%)  17 (9.8%) | p=.087 |
| Primary model of care^+^  Continuity (n=71)  Non-continuity/Rotational (n=142) | 59 (83.1%)  125 (88.0%) | 12 (16.9%)  17 (12.0%) | p=.323 |
| Experience in acute early pregnancy care^+^  None (n=89)  Prior or current (n=198) | 70 (78.7%)  181 (91.4%) | 19 (21.3%)  17 (8.6%) | **p=.003*** |

*****α=.05; **^**Valid percentages; **^^**Participant numbers may vary due to missing data; **^+^**Response categories condensed for analysis; **^#^**Selected response categories condensed for analysis

*Knowledge – care provision*

| Miscarriage care - All participants | Strong or fair knowledge  n (%^) | Limited or no knowledge  n (%^) | p-value |
| --- | --- | --- | --- |
| Whole sample (n^=327) | 225 (69%) | 102 (31%) | - |
| Professional group  Midwives (n=287)  Midwifery students (n=40) | 212 (73.9%)  13 (32.5%) | 75 (26.1%)  27 (67.5%) | **p=<.001*** |
| Location of practice^+^  Metropolitan (n=229)  Rural or remote (n=98) | 156 (68.1%)  69 (70.4%) | 73 (31.9%)  29 (29.6%) | p=.683 |
| Professional qualifications  Registered Nurse (RN) (n=173)  Not an RN (n=154) | 129 (74.6%)  96 (62.3%) | 44 (25.4%)  58 (37.7%) | **p=.017*** |
| Miscarriage care – Midwives only | **Strong or fair knowledge**  **n (%^)** | **Limited or no knowledge**  **n (%^)** | **p-value** |
| Professional qualifications^#^  Midwife and RN (n^=164)  Midwife but not RN (n^=123)  Endorsed midwife (n=70)  Midwife - not endorsed (n=217) | 124 (75.6%)  88 (71.5%)  53 (75.7%)  159 (73.3%) | 40 (24.4%)  35 (28.5%)  17 (24.3%)  58 (26.7%) | p=.438  p=.686 |
| Years of registration^+^  10 or less (n=114)  Greater than 10 years (n=173) | 71 (62.3%)  141 (81.5%) | 43 (37.7%)  32 (18.5%) | **p<.001*** |
| Primary model of care^#^  Continuity (n=71)  Non-continuity/Core or Rotational (n=142) | 48 (67.6%)  105 (73.9%) | 23 (32.4%)  37 (26.1%) | p=.332 |
| Experience in acute early pregnancy care^+^  None (n=89)  Prior or current (n=198) | 37 (41.6%)  175 (88.4%) | 52 (58.4%)  23 (11.6%) | **p=<.001*** |
| Ectopic pregnancy care – all participants | **Strong or fair knowledge**  **n (%^)** | **Limited or no knowledge**  **n (%^)** | **p-value** |
| Whole sample n^=327 | 189 (58%) | 138 (42%) | - |
| Professional group  Midwives (n=287)  Midwifery students (n=40) | 179 (62.4%)  10 (25.0%) | 108 (37.6%)  30 (75.0%) | **p=<.001*** |
| Location of practice^+^  Metropolitan (n=229)  Rural or remote (n=98) | 128 (55.9%)  61 (62.2%) | 101 (44.1%)  37 (37.8%) | p=.287 |
| Professional qualifications  Registered Nurse (n=173)  Not an RN (n=154) | 114 (65.9%)  75 (48.7%) | 59 (34.1%)  79 (51.3%) | **p=.002*** |
| Ectopic pregnancy care – Midwives only | **Strong or fair knowledge**  **n (%^)** | **Limited or no knowledge**  **n (%^)** | **p-value** |
| Professional qualifications^#^  Midwife and RN (n=164)  Midwife but not an RN (n=123)  Endorsed midwife (n=70)  Midwife but not endorsed (n=217) | 110 (67.1%)  69 (56.1%)  48 (68.6%)  131 (60.4%) | 54 (32.9%)  54 (43.9%)  22 (31.4%)  86 (39.6%) | p=.058  p=.218 |
| Years of registration^+^  10 or less (n=114)  Greater than 10 years (n=173) | 51 (44.7%)  128 (74.0%) | 63 (55.3%)  45 (26.0%) | **p=<.001*** |
| Primary model of care^#^  Continuity (n=71)  Non-continuity/Core or Rotational (n=142) | 40 (56.3%)  87 (61.3%) | 31 (43.7%)  55 (38.7%) | p= .489 |
| Experience in acute early pregnancy care^+^  None (n=89)  Prior or current (n=198) | 30 (33.7%)  149 (75.3%) | 59 (66.3%)  49 (24.7%) | **p=<.001*** |
| Hyperemesis gravidarum care – all participants | **Strong or fair knowledge**  **n (%^)** | **Limited or no knowledge**  **n (%^)** | **p-value** |
| Whole sample n^^=327 | 244 (75%) | 83 (25%) | - |
| Professional group  Midwives (n=287)  Midwifery students (n=40) | 231 (80.5%)  13 (32.5%) | 56 (19.5%)  27 (67.5%) | **p=<.001*** |
| Location of practice^+^  Metropolitan (n=229)  Rural or remote (n=98) | 170 (74.2%)  74 (75.5%) | 59 (25.8%)  24 (24.5%) | p=.808 |
| Professional qualifications^#^  Registered Nurse (n=173)  Not an RN (n=154) | 139 (80.3%)  105 (68.2%) | 34 (19.7%)  49 (31.8%) | **p=.012*** |
| Hyperemesis gravidarum care - Midwives only | **Strong knowledge**  **n (%^)** | **Limited knowledge**  **n (%^)** | **p-value** |
| Professional qualifications^#^  Midwife and RN (n=164)  Midwife but not an RN (n=123)  Endorsed midwife (n=70)  Midwife but not endorsed (n=217) | 134 (81.7%)  97 (78.9%)  55 (78.6%)  176 (81.1%) | 30 (18.3%)  26 (21.1%)  15 (21.4%)  41 (18.9%) | p=.547  p=.642 |
| Years of registration^+^  10 or less (n=114)  Greater than 10 years (n=173) | 82 (71.9%)  149 (86.1%) | 32 (28.1%)  24 (13.9%) | **p=.003*** |
| Primary model of care^#^  Continuity (n=71)  Non-continuity/Rotational (n=142) | 56 (78.9%)  111 (78.2%) | 15 (21.1%)  31 (21.8%) | p=.906 |
| Experience in acute early pregnancy care^+^  None (n=89)  Prior or current (n=198) | 55 (61.8%)  176 (88.9%) | 34 (38.2%)  22 (11.1%) | **p=<.001*** |

*****α=.05; **^**Valid percentages; **^^**Participant numbers may vary due to missing data; **^+^**Response categories condensed for analysis; **^#^**Selected response categories condensed for analysis
