# Supplementary material for: Exploring midwifery role and scope in acute early pregnancy care: a survey of midwives and midwifery students in Australia
Source: BMC Pregnancy Childbirth. 2025 Apr 16;25:458. doi: 10.1186/s12884-025-07567-3 (PMC12004735; doi:10.1186/s12884-025-07567-3)
Supplement: Supplementary file 3 — Supplementary Material 3 Additional file 3: Qualitative analysis exemplar: Process and application to participant’s comment [file 12884_2025_7567_MOESM3_ESM.docx]

**Additional file 3:** Qualitative content analysis process**^1-3^** and exemplar from data of one participant

| 1. PREPARE | 2. ORGANISE | | 3. REPORT |
| --- | --- | --- | --- |
| *Preparation and familiarisation →* | *Coding and*  *grouping data →* | *Condensing and ordering data →* | *Abstraction and*  *presentation of data* |
| Familiarisation with raw data - reading through several times  Analysis of *words or phrases* identified to  be appropriate for this study given nature of data (short answer responses)    Contextualise the data - consider the aim of the research as you become familiar with the content | Upload data into NVivo  Open **line-by-line coding** into initial codes with annotated notes in NVivo e.g., insights, possible relationships, highlighting  reflective quotes  **Preliminary grouping**  of initial codes into grouping matrix  **Pattern identification**  in the data | Use of **grouping matrix**  for categorisation of all relevant data/codes – iterative, dynamic back and forth rather than linear process  Identify/establish **connections** between categories  **Conceptual ordering** into hierarchy of generic and sub-categories | **Final condensing** of sub-categories → generic categories → main category  **Naming** of higher-order main categories using words that reflect the characteristics  of the content of the category  **Data visualisation** through interesting graphics that represent qualitative categories, to enhance and complement quantitative findings |
| Participant #87 | | | |
| *Raw data →* | *Initial NVivo codes →* | *Sub-categories →* | *Generic and main categories* |
| *‘It would be wonderful for women to have access in all health settings to a <20-week midwifery service.*  *I feel ED is wildly inappropriate for women losing a baby or having pregnancy related concerns (under 20 weeks).*  *Maternity settings should support a service separated from main maternity but still within maternity for midwives to support women who need us.*  *In the non-tertiary hospital I work in, we don’t see these women as midwives.*  *They are cared by ED staff/obstetric/gynae doctors...*  *…as a midwife I don’t hold a*  *lot of confidence in caring for women in this position, as I never have the opportunity*  *to do so.’* | ‘Access to midwives’  ‘Giving women choice’  ‘Creating early pregnancy services’  ‘The ED’  ‘Women’s experiences’  ‘The maternity setting’  ‘Separating care’  ‘Access to midwives’  ‘Creating early pregnancy services’  ‘Barriers for midwives’  ‘Access to midwives’  ‘Differences between health services’  ‘The ED’  ‘The MDT’  ‘Nurses’  ‘Understanding midwifery scope’  ‘Education and training’  ‘Pathways for qualified midwives’  ‘Barriers for midwives’ | Need to meet women’s emotional needs  Late contact with women in pregnancy  Potential for  midwives to provide comprehensive AEPC  The need for early engagement  Recognition that women have had poor experiences in settings such as ED  Women with AEP complications directed to gynaecology and ED settings  Believe midwives should provide acute early pregnancy care  Identifies need to meet women’s emotional needs  Maternity setting an unsuitable environment for some women with AEP complications  Women with AEP complications directed to gynaecology and ED settings  Lack of understanding and recognition of the role and scope of the midwife from other HCPs and HSPs  Limited midwifery presence in non-maternity settings  Lack of knowledge and experience  Lack of opportunities for students, graduates and midwives | Being ‘with woman’ regardless of gestation → **This IS midwifery scope**  Limited access to women in early pregnancy→ **Challenges for midwives**  Invested and interested in acute early pregnancy care→ **This IS midwifery scope**  Bring midwives and women together→ **Finding solutions**  The setting of care→ **Challenges for midwives**  Limited access to women in early pregnancy→ **Challenges for midwives**  Invested and interested in acute early pregnancy care→ **This IS midwifery scope**  Professional alignment of midwifery with acute early pregnancy care needs→ **This IS midwifery scope**  The setting of care→ **Challenges for midwives**  Limited access to women in early pregnancy→ **Challenges for midwives**  Inconsistent beliefs around midwifery role and scope→ **Challenges for midwives**  Limited access to women in early pregnancy→ **Challenges for midwives**  Limited educational and clinical exposure to AEPC→ **Challenges for midwives** |

AEPC: Acute early pregnancy care; ED: Emergency Department; AEP: Acute early pregnancy; HCPs: Health care professionals: MDT: Multidisciplinary team; HSPs: Health service providers

1. Neville S, Whitehead D. Analysing data in qualitative research. In: Whitehead D, Ferguson C, LoBiondo-Wood G, Haber J, editors. Nursing and midwifery research - methods and appraisal for evidence based practice. 6th ed. Chatswood, New South Wales: Elsevier; 2020. p. 136 - 55.

2. Elo S, Kyngäs H. The qualitative content analysis process. Journal of Advanced Nursing. 2008;62(1):107-15.

3. Rouder J, Saucier, O., Kinder, R., & Jans, M. What to Do With All Those Open-Ended Responses? Data Visualization Techniques for Survey Researchers Survey Practice. 2021.
